# Interacting Effects of Sea Louse (*Lepeophtheirus salmonis*) Infection and Formalin-Killed *Aeromonas salmonicida* on Atlantic Salmon Skin Transcriptome

**DOI:** 10.3389/fimmu.2022.804987

**Published:** 2022-03-24

**Authors:** Albert Caballero-Solares, Navaneethaiyer Umasuthan, Xi Xue, Tomer Katan, Surendra Kumar, Jillian D. Westcott, Zhiyu Chen, Mark D. Fast, Stanko Skugor, Richard G. Taylor, Matthew L. Rise

**Affiliations:** ^1^Department of Ocean Sciences, Memorial University, St. John’s, NL, Canada; ^2^Fisheries and Marine Institute, Memorial University, St. John’s, NL, Canada; ^3^Department of Pathology and Microbiology, Atlantic Veterinary College, University of Prince Edward Island, Charlottetown, PE, Canada; ^4^Cargill Aqua Nutrition, Cargill, Sea Lice Research Center (SLRC), Sandnes, Norway; ^5^Cargill Animal Nutrition and Health, Elk River, MN, United States

**Keywords:** Atlantic salmon, sea lice, formalin-killed bacterin, *Aeromonas salmonicida*, skin transcriptome

## Abstract

*Lepeophtheirus salmonis* (sea lice) and bacterial co-infection threatens wild and farmed Atlantic salmon performance and welfare. In the present study, pre-adult *L. salmonis*-infected and non-infected salmon were intraperitoneally injected with either formalin-killed *Aeromonas salmonicida* bacterin (ASAL) or phosphate-buffered saline (PBS). Dorsal skin samples from each injection/infection group (PBS/no lice, PBS/lice, ASAL/no lice, and ASAL/lice) were collected at 24 h post-injection and used for transcriptome profiling using a 44K salmonid microarray platform. Microarray results showed no clear inflammation gene expression signatures and revealed extensive gene repression effects by pre-adult lice (2,189 down and 345 up-regulated probes) in the PBS-injected salmon (PBS/lice *vs*. PBS/no lice), which involved basic cellular (e.g., RNA and protein metabolism) processes. Lice repressive effects were not observed within the group of ASAL-injected salmon (ASAL/lice *vs*. ASAL/no lice); on the contrary, the observed skin transcriptome changes –albeit of lesser magnitude (82 up and 1 down-regulated probes)– suggested the activation in key immune and wound healing processes (e.g., neutrophil degranulation, keratinocyte differentiation). The molecular skin response to ASAL was more intense in the lice-infected (ASAL/lice *vs*. PBS/lice; 272 up and 11 down-regulated probes) than in the non-infected fish (ASAL/no lice *vs*. PBS/no lice; 27 up-regulated probes). Regardless of lice infection, the skin’s response to ASAL was characterized by the putative activation of both antibacterial and wound healing pathways. The transcriptomic changes prompted by ASAL+lice co-stimulation (ASAL/lice *vs*. PBS/no lice; 1878 up and 3120 down-regulated probes) confirmed partial mitigation of lice repressive effects on fundamental cellular processes and the activation of pathways involved in innate (e.g., neutrophil degranulation) and adaptive immunity (e.g., antibody formation), as well as endothelial cell migration. The qPCR analyses evidenced immune-relevant genes co-stimulated by ASAL and lice in an additive (e.g., *mbl2b*, *bcl6*) and synergistic (e.g., *hampa*, *il4r*) manner. These results provided insight on the physiological response of the skin of *L. salmonis*-infected salmon 24 h after ASAL stimulation, which revealed immunostimulatory properties by the bacterin with potential applications in anti-lice treatments for aquaculture. As a simulated co-infection model, the present study also serves as a source of candidate gene biomarkers for sea lice and bacterial co-infection.

## Introduction

Aquaculture has been called upon to fill the predicted global fish demand-supply gap and nourish the growing human population with high-quality protein and health-promoting omega-3 fatty acids (1, 2). Atlantic salmon (*Salmo salar*) is one of the most important fish species farmed globally, both in biomass produced and market value; however, the success of Atlantic salmon aquaculture as a growing food-producing industry is threatened by disease outbreaks (3). The parasitic copepod *Lepeophtheirus salmonis* –commonly referred to as sea louse– is currently one of the main threats to Atlantic salmon aquaculture in the Northern hemisphere (4). The damage of lice outbreaks to the industry goes beyond production losses and the cost of anti-lice treatments [> US$500M only for Norway in 2015; > US$900M globally (5)]. Lice outbreaks at farm sites raise concerns about the welfare of the farmed and wild salmon and negatively influence the public perception of the aquaculture industry (6).

*L. salmonis* parasitizes a range of salmonids (genera *Salmo*, *Salvelinus*, and *Oncorhynchus*) to feed on their mucous, skin, and blood (7, 8). However, Atlantic salmon have been proven to be particularly susceptible to this parasitic infection (9, 10). The effectiveness of *L. salmonis* lies in its capacity to suppress Atlantic salmon’s skin inflammatory response during the early stages of the infection (11). During its development to adult, *L. salmonis* goes through 2 planktonic nauplii stages, a copepodid stage, 2 immobile chalimus stages, and 2 mobile pre-adult stages (7, 8). Failure to expel the juvenile sea lice allows them to continue feeding and develop to motile pre-adult and adult lice. The long duration of the infection and the higher degree of skin damage caused by pre-adult and adult *L. salmonis* further weakens Atlantic salmon, rendering them an easy target for secondary infections (11).

Co-infection of sea lice and pathogenic bacteria occurs naturally at Atlantic salmon sea cages (12). Co-infections can overwhelm the host’s immune defenses if the two pathogens do not antagonize one another, but rather interact synergistically (i.e., one pathogen increases host susceptibility to the other) (13). For instance, *L. salmonis* and *Moritella viscosa* –a Gram-negative bacterium causing winter ulcer disease in salmonids– co-infection hindered Atlantic salmon skin’s ability to heal and increased mortality rates compared with individuals infected with *M. viscosa* alone (14). The co-infection of *Caligus rogercresseyi* –the most prevalent parasitic copepod in Chile– and *Piscirickettsia salmonis* –a Gram-negative bacterium causing salmonid rickettsial septicemia (SRS)– is highly frequent in Chilean salmon farms and seems to be non-competitive (15), which may have severe implications in vaccines’ efficacy, and salmons’ performance and survival (16, 17).

The Gram-negative bacterium *Aeromonas salmonicida* (subspecies *salmonicida*) infects multiple internal organs and the skin of salmonids, causing furunculosis, a disease characterized by dermal furuncles and darkening, lethargy, and other mild clinical signs and low mortality rates in its chronic form; septicemia, necrotizing skin lesions, internal bleeding and sudden mass mortalities in its acute form (18). Due to its ubiquitousness among teleost species and environments and the significance of its impacts on fish farming operations (19), *A. salmonicida*-host (especially salmonids) interactions have increasingly been studied with the expansion of the aquaculture industry (20). Like *L. salmonis*, *A. salmonicida* virulence seems linked to its ability to immunosuppress the host (21). Only a few studies have investigated the interacting pathological effects of co-infection of *A. salmonicida* and a parasite (e.g., the ciliate *Philasterides dicentrarchi*) or virus [e.g., infectious pancreatic necrosis virus (IPNV)] on farmed fish (22, 23). However, the pathogenicity and virulence of their single infections call for the investigation of *L. salmonis* and *A. salmonicida* co-infection.

Previous transcriptomics studies have contributed to identifying the molecular processes underlying the physiological responses of the Atlantic salmon skin to sea lice infection (24–28), and Atlantic salmon and Atlantic cod internal organs to *A. salmonicida* infection and antigens (29–32). In contrast, our understanding of the Atlantic salmon skin’s global gene expression response to co-infections is just beginning but will aid in developing practical and integrative management strategies for aquaculture (e.g., clinical feeds, vaccines) to improve fish health.

The objective of the present study was to profile –for the first time– the Atlantic salmon skin transcriptome response to pre-adult *L. salmonis* infection in combination with an intraperitoneal injection of formalin-killed *A. salmonicida* bacterin. Identically prepared *A. salmonicida* bacterins had been used in previous studies to examine the innate immune response triggered in the spleen and head kidney of Atlantic cod (*Gadus morhua*) (29, 30). The inclusion of un-infected controls for both bacterin-treated and saline-treated salmon allowed for 1) assessing the modulatory effects of the *A. salmonicida* bacterin on the Atlantic salmon skin response to sea lice infection, and 2) analyzing the Atlantic salmon skin transcriptome response to *A. salmonicida* antigens, which had not been studied before. The consortium for Genomic Research on All Salmonids Project (cGRASP)-designed Agilent 44K salmonid oligonucleotide microarray (33) was the platform chosen for the present experiment given its proven reliability in providing robust Atlantic salmon transcriptomic data (34, 35).

## Materials and Methods

### Animals

Groups of 35 and 15 salmon smolts [238.9 ± 45.2 g; mean weight ± standard deviation (SD)] were –respectively– allocated in four 620-L tanks in the bio-containment zone at the Cold-Ocean Deep-Sea Research Facility (CDRF, Ocean Sciences Centre, Memorial University, NL, Canada) for the *Lepeophtheirus salmonis* challenge trial and two 620-L tanks at the Dr. Joe Brown Aquatic Research Building (JBARB, Ocean Sciences Centre) to serve as no-lice infection controls. For a detailed explanation of the fish acclimation process and holding conditions (e.g., flow-through water system), see Supplementary Methods. All procedures followed Canadian Council on Animal Care’s guidelines (approved Memorial University Institutional Animal Care Protocol 17-77-MR).

### Sea Lice Challenge

The salmon at CDRF were challenged with *L. salmonis* copepodids after an acclimation period of 79 days. As previously described (34), in preparation for lice exposure, water flow into the tanks was interrupted, and water volume was reduced by 50%. Oxygen was supplied to the water remaining in the tanks using air diffusers to prevent hypoxia. Then, sea lice copepodids were released into the tanks at a 50 lice/fish ratio and allowed to infect the salmon for 2 h. During the exposure, water dissolved oxygen levels (DO) and temperature were measured every 10 min. Any decrease in DO level during the challenge was quickly addressed by adjusting the air supply and remained above 7.1 mg/L and 72% saturation. No DO supersaturation occurred during the challenge. Water temperature increased by 0.4-0.6 °C on average. No mortalities were recorded. After the 2-h exposure period, the water supply was restored. For further details, see Supplementary Methods.

### Injection Challenge and Sample Collection

**Figure 1:**
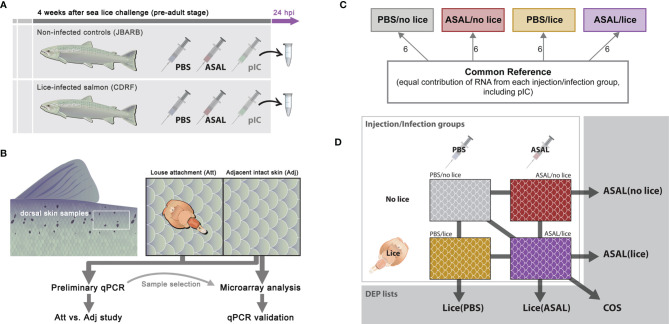
Experimental design of **(A)** Lice/ASAL co-stimulation trial, **(B)** skin sample collection on lice-infected salmon **(C)** 44K microarray experimental design, and **(D)** microarray data comparisons between injection/infection groups.

Four weeks after sea lice exposure, when lice were at the pre-adult stage, lice-infected salmon (CDRF) and non-infected salmon (JBARB) were fasted for 24 h and then subjected to an intraperitoneal (IP) injection of either phosphate-buffered saline (PBS; Gibco/ThermoFisher Scientific, Mississauga, ON, Canada), a solution of polyriboinosinic polyribocytidylic acid (pIC; 2 μg/μL; Sigma-Aldrich, Oakville, ON, Canada), or a suspension of formalin-killed *Aeromonas salmonicida* [ASAL; PBS-washed and pelleted commercial vaccine (Furogen dip, Novartis Canada, Charlottetown, PE, Canada), resuspended in PBS at an optical density of 1.0 at 600 nm wavelength (29)] (**Figure 1A**). For each tank at CDRF, 6 fish were injected with PBS, 6 with pIC, and 6 with ASAL at 1 μL/g of fish (wet mass). For each tank at JBARB, 4 fish were injected with PBS, 4-5 fish with pIC, and 4-5 fish with ASAL. At 24 h post-injection, salmon were euthanized by immersion in a seawater bath with 400 mg/L MS-222 (Syndel Laboratories, Vancouver, BC, Canada) and dissected for tissue sample collection. Two 1-cm^2^ dorsal skin samples were taken from every lice-infected salmon (CDRF): one sample around a louse attachment site and another sample from an adjacent intact skin area (i.e., no lice attached or damaged) (**Figure 1B**). Dorsal skin samples (also 1 cm^2^) from non-infected salmon (JBARB) were taken from the area directly posterior to the dorsal fin and dorsal to the lateral line. Skin samples were immediately flash-frozen with liquid nitrogen and stored at -80 °C until processed for RNA extraction. Total lice load was counted. Supplementary Methods contain additional information concerning pIC, ASAL preparations, and the fish handling and sampling procedures.

### RNA Extraction and Purification

Dorsal skin samples were homogenized in TRIzol reagent (Invitrogen/Life Technologies, Burlington, ON, Canada) with stainless steel beads (5 mm; QIAGEN, Mississauga, ON, Canada) using a TissueLyser II (QIAGEN), and subjected to RNA extraction following manufacturers’ instructions. Thirty micrograms of each total RNA sample were treated with 6.8 Kunitz units of DNaseI (RNase-Free DNase Set, QIAGEN) and then column-purified by using the RNeasy Mini Kit (QIAGEN) following the manufacturer’s instructions. The total RNA concentration and purity were assessed by ND-1000 UV spectrophotometry (NanoDrop, Wilmington, DE, USA), and the RNA integrity was examined by 1% agarose gel electrophoresis. RNA samples with tight 18S and 28S ribosomal RNA bands and high A260/280 and A260/230 ratios (> 1.8) were used in transcriptional analyses.

### Microarray Experimental Design

The present study included dorsal skin samples collected from the lice-infected salmon injected with PBS and ASAL (i.e., the salmon at CDRF; groups PBS/lice and ASAL/lice) and the non-infected salmon injected with PBS and ASAL (i.e., the salmon at JBARB; groups PBS/no lice and ASAL/no lice). Six biological replicates were allotted to each of the 4 injection/infection groups (i.e., 24 individual fish in total; **Figure 1C**).

This microarray study aimed to analyze the general skin transcriptome response to sea lice and ASAL co-stimulation, so it only used dorsal skin samples adjacent to louse attachment sites (**Figure 1B**). As explained in the section *Sample Selection for Microarray Analysis*, the present study included a complementary real-time quantitative polymerase chain reaction (qPCR) study comparing the mRNA levels of a selection of biomarker genes in the louse attachment (Att) and adjacent (Adj) skin sites (**Figure 1B**).

The microarray experiment followed Minimum Information About a Microarray Experiment (MIAME) guidelines (36), and it was conducted using cGRASP-designed Agilent 44K salmonid oligonucleotide microarrays [Gene Expression Omnibus (GEO) accession number: GPL11299 (33)]. The arrays were hybridized to anti-sense amplified RNA (aRNA) generated from high integrity and high purity skin total RNA. The analysis employed a common reference microarray experiment design, where the individual fish and the reference aRNA samples were labeled with different fluorescent dyes [Cy5 and Cy3 (GE HealthCare, Mississauga, ON, Canada), respectively]. For more details, see section *Microarray Hybridization and Data Acquisition*.

The common reference aRNA samples were prepared using pools of equal quantities of RNA from all 24 fish selected for the present study (i.e., PBS and ASAL-injected lice-infected and non-infected salmon; see section *Sample Selection for Microarray Analysis*), plus 12 additional RNA samples from non-infected (6 samples) and lice-infected (6 samples) salmon injected with pIC (**Figure 1C**). This design will allow future analyses of the shared molecular responses of Atlantic salmon dorsal skin to ASAL and pIC IP-injections, alone or in combination with lice infection.

### Sample Selection for Microarray Analysis

Biological variability associated with resistance to lice infection may interfere with the microarray analysis and lead to results that are not representative of the fish population under experimentation. Therefore, lice-infected fish (CDRF) with total lice counts below or above population average ± 1 SD were not included in the microarray analysis. A total of 7 PBS/lice and 7 ASAL/lice salmon did not comply with this criterion and were not included in the study. In addition, in the interest of sample standardization, only lice-infected salmon that provided intact dorsal skin samples adjacent to an attached louse were considered. Among the excluded lice-infected salmon, there were 6 PBS/lice and 1 ASAL/lice that had intact skin samples taken next to 2 attached lice, and 3 PBS/lice and 8 ASAL/lice that did not present louse attachment and intact adjacent sites on their dorsal skin suitable for sample collection. Eight biological replicates for the PBS/lice and ASAL/lice groups passed sample filtering.

The RNA samples of the infected salmon that passed filtering were subjected to qPCR analysis (**Figure 1B**). The transcript levels of well-known inflammation [*interleukin 1 beta* (*il1b*) and *cyclooxygenase-2* (*cox2*)], acute-phase response (APR) [*serum amyloid A-5 protein* (*saa5*)], tissue remodeling [*matrix metallopeptidase 13 A* (*mmp13a*)], and anti-bacterial [*toll-like receptor 5 A, soluble* (*tlr5a*)] gene biomarkers were qPCR-quantified on Att and Adj skin samples. The methodology for primer design and quality testing, normalizer selection, cDNA synthesis, and qPCR analysis are explained in the *qPCR Analyses* section and the Supplementary Methods. The obtained qPCR data were analyzed *via* Principal Component Analysis (PCA; see *Statistical Analysis* section) to select the 6 most representative biological replicates (i.e., closely clustered in the multivariate space) for each of the 4 injection/infection groups. The gene expression results arising from these analyses have been added here as a complementary qPCR study comparing Att and Adj gene expression signatures.

The 6 biological replicates in the microarray analysis representing the non-infected salmon (JBARB) were randomly selected from 8 PBS/no lice salmon and 9 ASAL/no lice salmon. Lice-infected salmon that passed sample filtering and all non-infected salmon were considered in the qPCR confirmation of the microarray results.

### Microarray Hybridization and Data Acquisition

One microgram of DNaseI-treated and column-purified RNA from each individual fish and the common reference pool was *in vitro*-transcribed into aRNA using the Amino Allyl MessageAmp™ II aRNA Amplification Kit (Ambion, ThermoFisher Scientific, Waltham, MA, USA), following the manufacturer’s instructions. The resulting aRNAs were quality-checked and quantified using agarose gel electrophoresis and ND-1000 UV spectrophotometry (NanoDrop). Twenty micrograms of each aRNA sample were precipitated overnight using a standard ethanol precipitation method and re-suspended in coupling buffer (Ambion). Common reference and individual fish aRNAs were labeled with Cy3 and Cy5, respectively, following the manufacturer’s instructions. The labeled aRNA concentration and labeling efficiency were measured using the microarray feature in the ND-1000 UV spectrophotometer. For each array, an equal quantity (825 ng) of an individual fish Cy5-labeled and reference Cy3-labeled aRNA were fragmented and co-hybridized to a 44K microarray at 65°C for 17 h with rotation (10 rpm) using an Agilent hybridization oven. The array slides were washed immediately after hybridization as per the manufacturer’s instructions and dried by centrifuging at 200 × g for 5 min at room temperature.

Microarray slides were immediately scanned at 5-μm resolution using a SureScan Microarray Scanner System (Agilent) and Microarray Scan Control Software v.9.1 following the built-in Agilent HD 2-color gene expression microarray scan protocol. The signal intensity data were extracted and subjected to linear and LOESS normalization using Agilent Feature Extraction Software v12.0 (Agilent). Probes of low quality (e.g., signal not above background) or with absent values in more than 25% of all 24 arrays were removed from the dataset, and the missing values were imputed using GeneSpring Software v14.9 (Agilent). The final dataset of normalized log_2_-transformed Cy5/Cy3 ratios consisted of 25,882 probes for all arrays (GEO accession number: GSE186292; https://www.ncbi.nlm.nih.gov/geo/query/acc.cgi?acc=GSE186292).

### Microarray Data Analysis

Normalized log_2_-transformed ratios were analyzed *via* Significance Analysis of Microarrays (SAM) (37) to identify differentially expressed probes (DEPs) between injection/infection groups at a false discovery rate (FDR) of 5% using the Bioconductor R package *siggenes* (38). Five SAM comparisons were made between the different injection/infection groups (**Figure 1D**). The comparison between ASAL/lice and PBS/lice was meant to explore the skin transcriptome response to ASAL in the lice-infected fish [ASAL(lice) DEP list]. The comparison ASAL/no lice *vs.* PBS/no lice covered the skin transcriptome response to ASAL in the non-infected fish [ASAL(no lice) list]. The PBS/lice *vs.* PBS/no lice comparison searched for lice-responsive probes in PBS-injected salmon [Lice(PBS) list]. The ASAL/lice *vs.* ASAL/no lice comparison searched for lice-responsive probes in ASAL-injected salmon [Lice(ASAL) list]. The comparison ASAL/lice *vs.* PBS/no lice aimed to identify probes responsive to ASAL and lice infection (i.e., co-stimulated DEPs; COS list).

For gene identification in the DEP lists, a previous annotation of the 44K 60mer oligonucleotide probes (39) was updated *via* BLASTx searches of the contiguous sequences (contigs) used to design the probes against the NCBI non-redundant amino acid (nr) and Swiss-Prot databases (thresholds: E-value < 1e-5, identity percentage > 75%, query coverage >50%). BLASTn searches using the 60mer probes [against the NCBI non-redundant nucleotide (nt) database] were conducted to verify the annotation of the updated probes (threshold: ≤ 2 mismatches; no alignment gaps allowed). Human gene symbols were assigned to the annotated probes based on HUGO Gene Nomenclature Committee (HGNC; https://www.genenames.org/) and/or GeneCards (https://www.genecards.org/) databases.

### Network and Gene Ontology Enrichment Analyses

Gene ontology (GO) term enrichment analyses (GTEA) were conducted for each DEP list using ClueGO (40) plugin in Cytoscape (v3.5.1) (41). This analysis disregarded DEP redundancy (i.e., multiple probes annotated as the same gene); it only considered the differentially expressed genes (DEGs) putatively represented by the DEP lists. Right-sided hypergeometric tests (i.e., for GO term over-representation) were performed using the human GO database (UniProt: 27.02.2019) for Biological Processes (BPs), with an adjusted p-value cut-off level (Benjamini-Hochberg test) of 0.05. The entire 44K salmon array was used as the reference gene list. ClueGO linked the over-represented GO terms using kappa statistics (42), thus generating GO term networks. The kappa coefficient threshold for the analysis was 0.4. The relative frequency of up-regulated and down-regulated DEGs was used to calculate the z-score (43) of each GO term arising from the GTEA. The over-represented GO terms were classified, using Gene Ontology Browser (http://www.informatics.jax.org), into 4 functional themes: 1) metabolic processes; 2) cellular processes; 3) immune/stress processes; and 4) development/healing processes. Some GO groups comprised terms from different themes; in such cases, the group is colored according to the theme with the highest number of GO terms. Additional information on kappa statistics and GO term classification criterion can be found in the Supplementary Methods.

### qPCR Analyses

Forty-one microarray-identified genes of interest (GOIs) were qPCR-analyzed to confirm the microarray results (see *Statistical Analyses* for more information). Despite not being microarray-identified, the qPCR confirmation study also included *tlr5a* to better represent bacterial recognition processes.

First-strand cDNA synthesis and qPCR amplifications were performed following Minimum Information for Publication of qPCR Experiments [MIQE (44)]-compliant methods previously published (34, 35) and described in the Supplementary Methods. A ViiA 7 Real-Time PCR system (Applied Biosystems/Life Technologies, Foster City, CA, USA) was used for the qPCR experiments. Primer pairs were either designed or selected from previous studies, and quality-tested [e.g., single-product amplification, efficiency (45)] as described in Caballero-Solares et al. (46) and Supplementary Methods. All information concerning primer sequences and quality-check results is shown in the **Supplemental Table S1**.

Five candidate normalizer genes were tested for mRNA level stability across injection/infection groups. These genes were *60S ribosomal protein L32* (*rpl32*), *elongation factor 1-alpha 1* (*ef1a1*), *polyadenylate-binding protein, cytoplasmic 1* (*pabpc1*), *eukaryotic translation initiation factor 3 subunit D* (*eif3d*), *ATP binding cassette sub-family f member 2* (*abcf2*). These candidate normalizer genes were selected based on previous experience with infected or pathogen-associated molecular patterns (PAMP)-challenged Atlantic salmon (35, 47). *rpl32* and *pabpc1* were chosen as the most stably expressed based on geNorm analyses [M-values 0.160 and 0.158, respectively; qBASE plus, Biogazelle NV, Belgium (48)].

The relative quantity (RQ) of each qPCR-analyzed GOI was calculated using a qBase relative quantification framework (49, 50) through normalization to *rpl32* and *pabpc1*, with amplification efficiencies incorporated. The RQ values of each GOI were calibrated to the sample that had the lowest normalized gene expression (i.e., assigned an RQ value = 1.0).

### Statistical Analyses

Microarray data were subjected to Pearson correlation tests to identify significant relationships between expression levels and total lice load. Non-infected salmon were not considered for the correlation analyses. As in previous studies (9, 20, 21), the validity of the microarray results was assessed by a linear regression analysis of qPCR and microarray-derived log_2_-transformed fold-changes. Gene expression fold-changes were calculated following the formula 2^A-B^, A and B being the RQs of two different injection/infection groups (e.g., ASAL/lice *vs*. PBS/no lice) (51).

Total lice load counts were analyzed for PBS/ASAL injection effects using Mann-Whitney U test as the data failed to comply with the normality assumption (Shapiro-Wilk test). Changes in the transcript levels of the qPCR-analyzed genes were modeled using generalized linear models (GLMs). For the qPCR confirmation experiment, the factors tested were ASAL treatment (i.e., PBS/ASAL injection) and *L. salmonis* infection (i.e., presence/absence). For the complementary qPCR experiment (arising from the preliminary analyses conducted for sample selection), the factors tested were ASAL treatment (i.e., PBS/ASAL injection) and skin site (i.e., Adj/Att). Once modeled, we tested the significance of each factor and the interactions between factors through ANOVA. Pairwise comparisons between injection/infection groups were carried out using estimated marginal means (EMMs). Similar to the microarray data, the qPCR-confirmation results were analyzed for correlation with total lice load counts (Pearson correlation test). Again, non-infected salmon were not considered for the correlation analyses. The microarray and qPCR confirmation experiment datasets were analyzed using Principal Component Analysis (PCA). For the qPCR data-based PCA, the scores of the first two principal components were also subjected to the same statistical analyses as the qPCR confirmation data (i.e., GLMs for lice and ASAL effects; EMMs for inter-group pairwise comparisons).

All statistical analyses –except for GTEA– were conducted using the R environment, more specifically the packages: *glm* (generalized linear models), *car* (one-way ANOVA), *emmeans* (estimated marginal means), *corrplot* (Pearson correlation), *factoextra* and *ade4* (PCA). Results were plotted using the R packages *ggplot2* and *ggpubr*. The statistical significance threshold was p-value (*p*) <0.05 for all statistical analyses.

## Results

### Lice Infection Levels

The entire group of lice-infected salmon (i.e., PBS and ASAL-injected; n = 48) showed an average total lice load of 12.0 ± 5.8 (SD). There were no significant differences in total lice load counts between PBS and ASAL-injected salmon (Mann-Whitney U test; *p* = 0.985).

### Microarray Results

**Figure 2:**
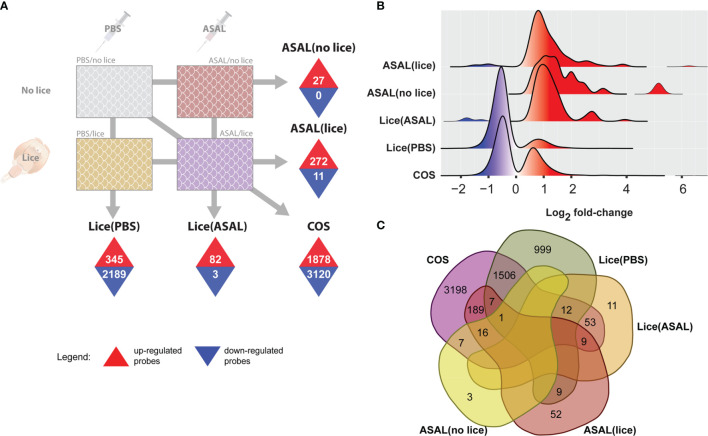
Summary of microarray results. **(A)** Identified differentially expressed probes (DEPs) using SAM (5% FDR). Numbers inside upward pointing triangles represent the number of up-regulated DEPs; those inside downward pointing triangles represent the number of down-regulated DEPs. **(B)** Histogram of the frequency density of log_2_-transformed fold-changes for the different DEPs lists. Tones of red and blue colors indicate up- and down-regulation, respectively. **(C)** Venn diagram showing the total number of exclusive and overlapped DEPs among lists.

SAM (5% FDR) identified 345 up-regulated and 2,189 down-regulated DEPs in the comparison PBS/lice *vs*. PBS/no lice [i.e., Lice(PBS) list; **Figure 2A** and **Supplemental Table S2**], and 82 up-regulated and 3 down-regulated DEPs in the comparison ASAL/lice *vs.* ASAL/no lice [i.e., Lice(ASAL) list]. The comparison ASAL/lice *vs.* PBS/lice [i.e., ASAL(lice) list] identified 272 up-regulated and 11 down-regulated DEPs, whereas ASAL/no lice *vs.* PBS/no lice [i.e., ASAL(no lice) list] revealed 27 up-regulated DEPs. The skin transcriptome differences between the co-stimulated salmon (i.e., ASAL/lice) and PBS/no lice salmon accounted for 1,878 up-regulated and 3,120 down-regulated DEPs (i.e., COS list). The PCA of the complete microarray dataset showed segregation among the different injection/infection groups in the multivariate space (**Supplementary Figure S1**). The distance between groups reflected the size of their corresponding DEP list; for example, the largest DEP list (i.e., COS) derived from the two most distant groups in the PCA (i.e., ASAL/lice and PBS/lice).

In all DEP lists, the majority of up-regulated probes showed moderate fold-changes [i.e., < 2 log_2_ fold-change (FC)], although the distribution of the complete lists stretched towards high induction levels (i.e., above 4 log_2_ FC; **Figure 2B**). Up-regulated DEPs in ASAL(lice), ASAL(no lice), and Lice(ASAL) had multimodal log_2_ FC distributions, with a predominant peak close to 1 log_2_ FC for ASAL(no lice) and Lice(ASAL), and slightly below 1 log_2_ FC for ASAL(lice). FCs of the up-regulated DEPs in Lice(PBS) and COS lists displayed a single peak below 1 log_2_ FC. Down-regulated DEPs in Lice(PBS) and COS characteristically showed mild log_2_ FCs above -1, whereas in Lice(ASAL) and ASAL(lice), they presented some log_2_ FCs below -1. ASAL(no lice) presented no down-regulated probes.

Of the 4,998 DEPs in the COS list (i.e., 1,878 up + 3,120 down), 3,198 (64% of the total) were COS-exclusive and 1,800 (36%) were shared with other lists (**Figure 2C** and **Supplemental Table S2**). Within the shared DEPs, 1,526 were also found in Lice(PBS), 222 in ASAL(lice), 74 in Lice(ASAL), and 24 in ASAL(no lice). Lice(PBS) list comprised 999 exclusive DEPs (39% of the total) and 1,535 DEPs (61%) shared with other lists. Lice(ASAL) had 11 (13%) exclusive and 74 (87%) shared DEPs. ASAL(lice) presented 52 (18%) exclusive and 231 (82%) shared DEPs. ASAL(no lice) list was composed of 3 (11%) exclusive and 24 (89%) shared DEPs. No DEPs were shared between Lice(ASAL) and ASAL(no lice) lists.

### Functional Analysis of the Skin Transcriptome Responses

**Figure 3:**
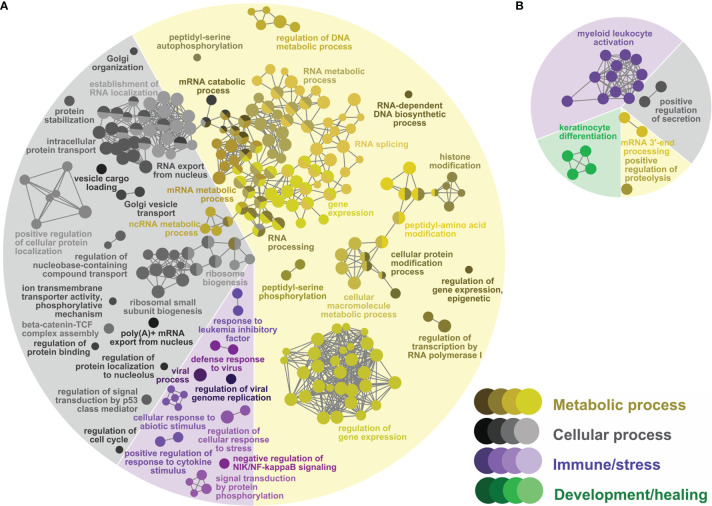
Network GO term enrichment analysis of the differentially expressed genes (DEGs) in **(A)** the Lice(PBS) list, and **(B)** the Lice(ASAL) list. Nodes represent over-represented GO terms (right-sided hypergeometric test; adjusted p-value < 0.05). Nodes are colored according to their assigned functional theme. Highly related terms (kappa coefficient > 0.4) are connected with grey lines. Single GO terms (i.e., single node) and GO networks are grouped and colored by functional theme (e.g., metabolic process, immune/stress) and arranged to fit the pie chart sectors representing the proportion of GO terms in each functional theme. Some GO groups comprised terms from different themes; in such cases, the group is colored according to the theme with the highest number of GO terms.

The GTEA found 230 over-represented biological process GO terms (**Figure 3A** and **Supplemental Table S3**) in the Lice(PBS) list: 134 (58%) metabolic processes, 75 (33%) cellular processes, and 21 (9%) immune/stress processes. The over-represented metabolic processes in Lice(PBS) focused on nucleic acid (e.g., “mRNA metabolic process”) and protein metabolism (e.g., “protein modification process”). The over-represented cellular processes in Lice(PBS) included organelle organization and biogenesis (e.g., “ribosome biogenesis”), RNA and protein localization and transport (e.g., “intracellular protein transport”), and the regulation of cell signaling (e.g., “regulation of signal transduction by p53 class mediator”) and cell cycle (e.g., “regulation of cell cycle”). Several over-represented immune/stress processes in Lice(PBS) were related to viral infection (e.g., “defense response to virus”); others were related to responses to cytokines (e.g., “positive regulation of response to cytokine stimulus”), and different abiotic stressors (e.g., “cellular response to abiotic stimulus”).

In the Lice(ASAL) list, mostly immune/stress and development/healing processes were over-represented [9 (43% of all 21) and 4 (19%) processes, respectively; **Figure 3B** and **Supplemental Table S4**]. All immune/stress processes were related to neutrophil-mediated immunity (i.e., “myeloid leukocyte activation”), whereas all development/healing processes were related to skin development (i.e., “skin development”). The 5 over-represented cellular processes (24%) were grouped with the neutrophil-mediated immunity-related processes [e.g., “exocytosis” in group 4 (**Supplemental Table**]. The 3 over-represented metabolic processes included proteolysis (e.g., “positive regulation of proteolysis”) and RNA 3’-end processing (e.g., “mRNA 3’-end processing”).

**Figure 4:**
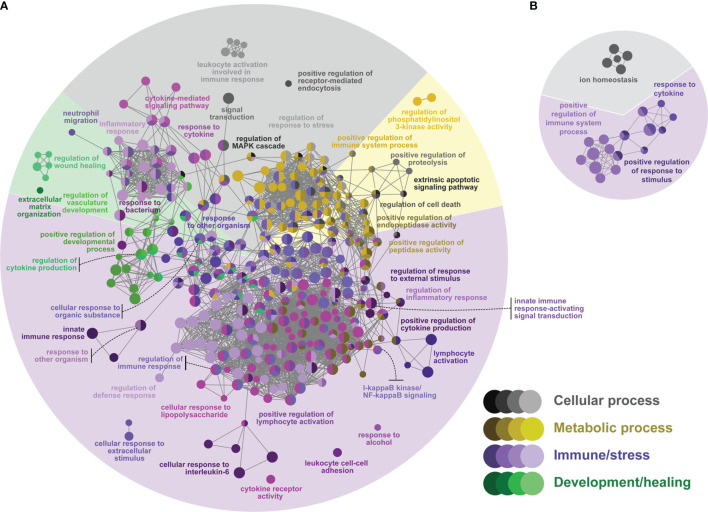
Network GO term enrichment analysis of the differentially expressed genes (DEGs) in **(A)** the ASAL(lice) list, and **(B)** the ASAL(no lice) list. Nodes represent over-represented GO terms (right-sided hypergeometric test; adjusted p-value < 0.05). Nodes are colored according to their assigned functional theme. Highly related terms (kappa coefficient > 0.4) are connected with grey lines. Single GO terms (i.e., single node) and GO networks are grouped and colored by functional theme (e.g., metabolic process, immune/stress) and arranged to fit the pie chart sectors representing the proportion of GO terms in each functional theme. Some GO groups comprised terms from different themes; in such cases, the group is colored according to the theme with the highest number of GO terms.

ASAL(lice) presented 77 (26%) and 173 (58%) over-represented cell and immune/stress processes, respectively (**Figure 4A** and **Supplemental Table S5**), whereas ASAL(no lice) had 8 over-represented cell processes (36% of all 22) and 14 immune/stress processes (64%) (**Figure 4B** and **Supplemental Table S6**). In ASAL(lice), there were cellular processes involved in endocytosis and apoptosis (e.g., “positive regulation of receptor-mediated endocytosis” and “regulation of cell death”, respectively). Some cell processes in ASAL(no lice) were related to cellular ion homeostasis (e.g., “ion homeostasis”). Many cellular processes in ASAL(lice) and ASAL(no lice) were grouped with processes of different themes (e.g., immune/stress processes, development/healing processes) and spanned over various cell signaling pathways, such as the MAPK/ERK pathway [e.g., “regulation of MAPK cascade”, group 7 of the ASAL(lice) list (**Supplemental Table S5**); “signal transduction”, groups 0 and 7 of the ASAL(no lice) list (**Supplemental Table S6**)], exocytosis [e.g., “secretion by cell”, group 16 of the ASAL(lice) list (**Supplemental Table S5**)], and cell chemotaxis [e.g., “cell migration”, group 32 of the ASAL(lice) list (**Supplemental Table S6**)]. Other immune/stress processes over-represented in ASAL(lice) were related to anti-bacterial responses (e.g., “response to bacterium”), inflammatory response (e.g., “I-kappaB kinase/NF-kappaB signaling”), and lymphocyte activation (e.g., “positive regulation of lymphocyte activation”). Also, ASAL(lice) had over-represented metabolic and development/healing processes, whereas ASAL(no lice) did not (**Figure 4B**). Most over-represented metabolic processes in ASAL(lice) were associated with proteolysis and regulation of endopeptidase activity (e.g., “positive regulation of proteolysis”). Over-represented development/healing processes in ASAL(lice) related to wound healing and hemostasis (e.g., “regulation of wound healing”), angiogenesis (e.g., “regulation of vasculature development”), and extracellular matrix (ECM) organization (e.g., “extracellular matrix organization”) (**Figure 4A**).

**Figure 5:**
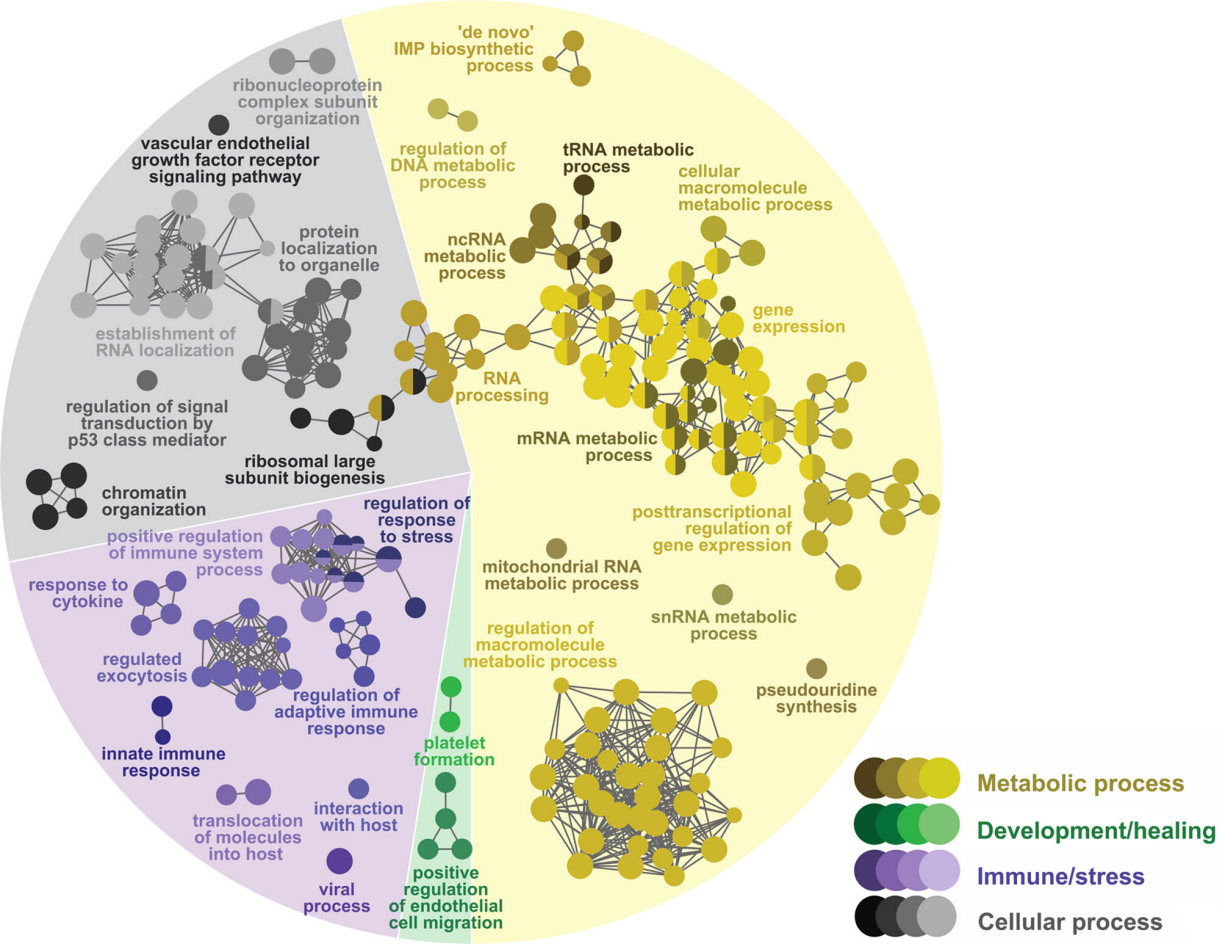
Network GO term enrichment analysis of the differentially expressed genes (DEGs) in the COS list. Nodes represent over-represented GO terms (right-sided hypergeometric test; adjusted p-value < 0.05). Nodes are colored according to their assigned functional theme. Highly related terms (kappa coefficient > 0.4) are connected with grey lines. Single GO terms (i.e., single node) and GO networks are grouped and colored by functional theme (e.g., metabolic process, immune/stress) and arranged to fit the pie chart sectors representing the proportion of GO terms in each functional theme. Some GO groups comprised terms from different themes; in such cases, the group is colored according to the theme with the highest number of GO terms.

The GTEA found 223 biological processes over-represented by the COS list (**Figure 5** and **Supplemental Table S7**): 116 (52%) were classified as metabolic processes, 55 (25%) as cellular processes, 45 (20%) as immune/stress processes, and 7 (3%) as development/healing processes. Similar to Lice(PBS), most over-represented metabolic processes in the COS list were directly or indirectly related to the metabolism of nucleic acids (e.g., mRNA, ncRNA, DNA) and proteins. Further, the cellular processes spanned over cell organelle organization and biogenesis (e.g., “ribosomal large subunit biogenesis”), RNA and protein localization and transport (e.g., “establishment of RNA localization”), cell signaling (e.g., “regulation of signal transduction by p53 class mediator”), and cell cycle regulation (e.g., “regulation of cell cycle G1/S phase transition”). Among the over-represented immune/stress processes, there were many related to innate and adaptive immune responses (e.g., groups 19 and 27, led by “innate immune response” and “regulation of adaptive immune response”, respectively). Also, there were processes related to viral infection (e.g., “viral process”), neutrophil-mediated immune processes (e.g., “regulated exocytosis”), and response to stress (e.g., “regulation of response to stress”). The development/healing processes involved platelet formation (i.e., group 21, led by “platelet formation”) and endothelial cell migration (e.g., “positive regulation of endothelial cell migration”).

**Figure 6:**
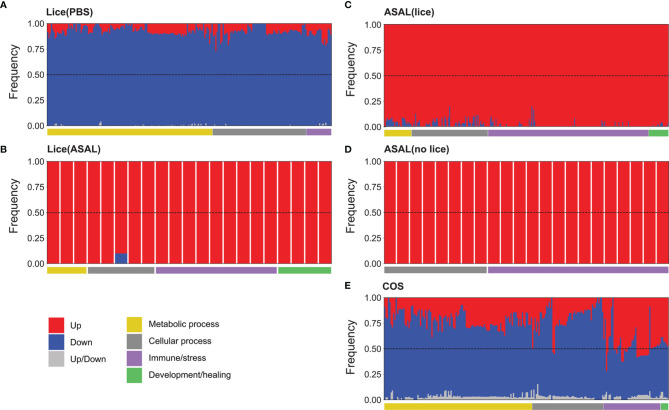
Stacked bar plots showing the relative frequency of differentially expressed genes (DEGs) detected by up-regulated (red) and down-regulated (blue) microarray probes or by both up and down-regulated probes (light grey) for the GO terms over-represented in the **(A)** Lice(PBS), **(B)** Lice(ASAL), **(C)** ASAL(lice), **(D)** ASAL(no lice), and **(E)** COS lists. The GO terms are arranged based on their functional theme (e.g., metabolic process, immune/stress; see horizontal bars below the stacked bar plots).

Regardless of the theme, down-regulated DEGs were predominant in all the over-represented biological processes of the Lice(PBS) list (**Figure 6A**). Conversely, all biological processes of the Lice(ASAL), ASAL(no lice), and ASAL(lice) were mostly or exclusively represented by up-regulated DEGs (**Figures 6B–D**).

Metabolic and cellular processes in the COS list were predominantly represented by down-regulated genes, except for 2 cellular processes: “regulated exocytosis” and “exocytosis” (both in group 32, together with several immune/stress processes), which were represented by up-regulated genes in a slightly higher proportion than down-regulated genes (**Figure 6E**; for details see **Supplemental Table S7**). The up-regulated:down-regulated DEG ratio varied among groups of immune/stress processes. For example, processes involved in viral infection, parasite-host interaction, and regulation of stress response were represented by down-regulated genes mostly. On the other hand, the proportion of up-regulated genes was over that of down-regulated in processes related to, e.g., antigen processing and presentation, cytokine production, innate immune response, negative regulation of adaptive immune response, and neutrophil activation. Development/healing processes related to platelet formation and angiogenesis had somewhat more down-regulated than up-regulated representative DEGs, whereas “vascular endothelial growth factor receptor signaling pathway” was represented by a slightly higher number of up-regulated DEGs (**Figure 6E** and **Supplemental Table S7**).

### qPCR Analysis of Microarray-Identified Transcripts

The log_2_ FCs calculated using the qPCR data of the same individuals selected for microarray analysis were significantly linearly correlated with the microarray log_2_ FCs [**Supplementary Figure S2** (‘selected samples’ linear regression model); *r^2^* = 0.838]. Adding more biological replicates to the qPCR log_2_ FC calculation decreased the correlation with the microarray log_2_ FCs [**Supplementary Figure S2** (‘all samples’ linear regression model); *r^2^* = 0.703], but the linear regression model remained highly significant (*p* < 0.0001).

**Figure 7:**
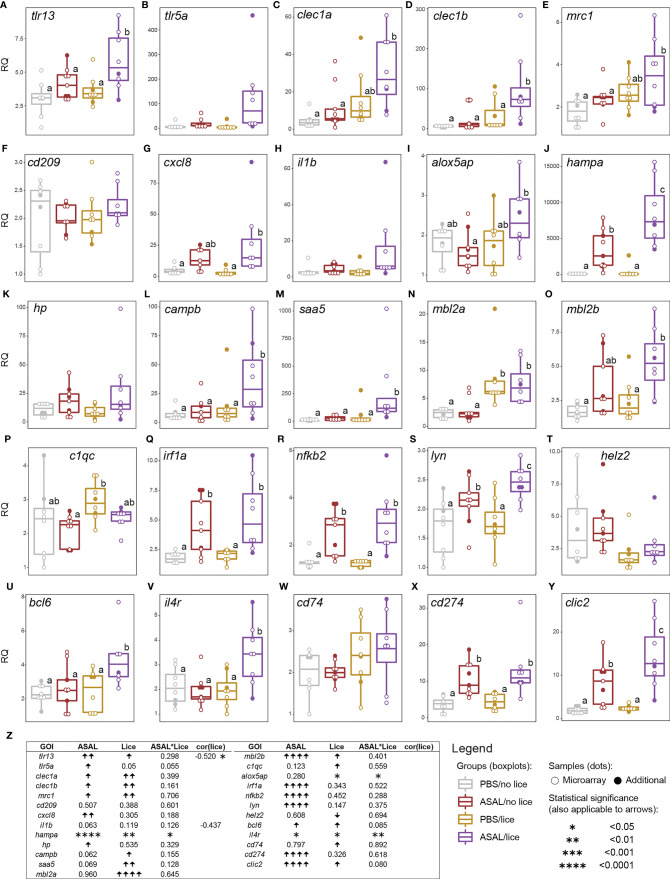
qPCR results of lice, ASAL, and lice+ASAL-responsive genes putatively involved in **(A–F)** pathogen/damage-associated molecular pattern recognition, **(G–I)** inflammatory responses, **(J–P)** innate immune responses, **(Q–U)** transcriptional regulation in innate and adaptive immune responses, and **(V–Y)** adaptive immunity-related processes. qPCR data are represented with scatter plot/boxplot overlays (n = 8-9 per injection/infection group). The scatter plot differentiates the additional biological replicates included in the qPCR validation (solid dots) from those selected for microarray analysis (empty dots). Lowercase letters indicate significant differences between groups, as determined by estimated marginal means. **(Z)** Summary of the results from the generalized linear model (GLM) analysis of the qPCR data and Pearson coefficients (*r*) for significant gene transcript levels and total lice count correlations. Upward and downward arrows indicate significant up and down-regulation, respectively. Asterisks are used instead of arrows when a significant lice and ASAL interaction (i.e., lice*ASAL) was detected. Asterisks also indicate a significant Pearson correlation. The statistical significance threshold was *p < *0.05 for all statistical analyses. *il1b*’s Pearson coefficient is indicated due to its closeness to statistical significance (i.e., 0.05 < *p* < 0.10) and physiological relevance.

The co-stimulated salmon (i.e., ASAL/lice) showed higher transcript levels of the putatively immune-related GOIs *toll-like receptor 13* (*tlr13*), *C-type lectin domain family 1 member B* (*clec1b*)*, hepcidin antimicrobial peptide A* (*hampa*)*, cathelicidin antimicrobial peptide B* (*campb*)*, saa5, tyrosine-protein kinase Lyn* (*lyn*)*, B-cell lymphoma 6 protein* (*bcl6*)*, interleukin 4 receptor* (*il4r*), and *chloride intracellular channel 2* (*clic2*) than the other injection/infection groups (EMM pairwise comparisons; **Figures 7A, D, J, L, M, S, U, V, Y**). The mRNA levels of *C-type lectin domain family 1 member A* (*clec1a*) and *mannose receptor, C type 1* (*mrc1*) were higher in the ASAL/lice salmon than in the non-infected salmon (i.e., PBS/no lice and ASAL/no lice; **Figures 7C, E**). *Interleukin-8* (*cxcl8*) and *mannose binding lectin 2 B* (*mbl2b*) had higher expression levels in the ASAL/lice salmon than in the PBS-treated salmon (i.e., PBS/no lice and PBS/lice; **Figures 7G, O**). ASAL/lice salmon had higher *arachidonate 5-lipoxygenase activating protein* (*alox5ap*) mRNA levels than ASAL/no lice salmon (**Figure 7I**). These patterns result from the additive (i.e., for *tlr13, clec1a, clec1b, mrc1, cxcl8, mbl2b, bcl6,* and *clic2*) and synergistic (i.e., stronger effects than with the sum of the individual factors; for *alox5ap, hampa* and *il4r*) effects of ASAL injection and lice infection (GLM results; **Figure 7Z**). For *tlr5a, il1b, campb,* and *saa5*, GLM analyses showed close to significant effects (i.e., 0.05 < p < 0.1; **Figure 7Z**) for one of the stimuli: ASAL (i.e., *campb, saa5, il1b*) or lice (i.e., *tlr5a*). Pairwise comparisons suggest ASAL+lice additive effects on *lyn* mRNA levels (**Figure 7S**), but the GLM results were not significant for lice (p = 0.147; **Figure 7Z**). *tlr5a* showed an overall ASAL induction (**Figure 7Z**), but no significant pairwise differences were found between groups (**Figure 7B**). Regarding the single-stimulus exclusively responsive GOIs, ASAL up-regulated *haptoglobin* (*hp*), *interferon regulatory factor 1 A* (*irf1a*), *nuclear factor kappa B subunit 2* (*nfkb2*), and *programmed cell death 1 ligand 1* (*cd274*), regardless of lice infection (**Figures 7K, Q, R, X, Z**). ASAL alone (i.e., ASAL/no lice) up-regulated *hampa, lyn*, and *clic2* compared with the PBS-treated salmon (**Figures 7J, S, Y**). On the other hand, lice infection up-regulated *mannose binding lectin 2 A* (*mbl2a*) and *HLA class II histocompatibility antigen gamma chain* (*cd74*), and down-regulated *helicase with zinc finger 2* (*helz2*), regardless of ASAL treatment (**Figures 7N, T, W, Z**). The *complement C1q C chain* (*c1qc*) transcript levels were higher in PBS/lice salmon than in ASAL/no lice salmon (**Figure 7P**). Total lice load counts were significantly negatively correlated with *tlr13* (**Figure 7Z**) and close-to-significantly negatively correlated with *il1b* (p = 0.091; **Figure 7Z**).

**Figure 8:**
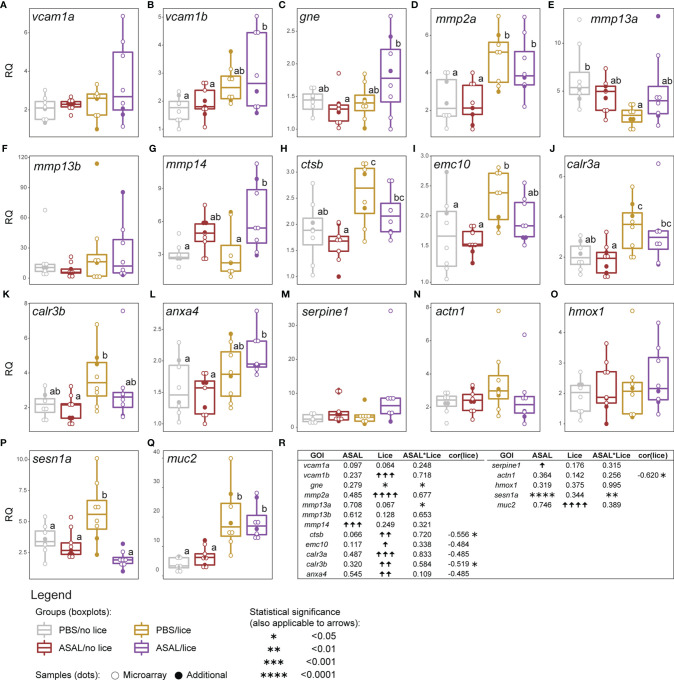
qPCR results of lice, ASAL, and lice+ASAL-responsive genes putatively involved in **(A–C)** cell adhesion, **(D–L)** tissue remodeling and development, **(M, N)** hemostasis and platelet activation, **(O, P)** heme degradation and protection against oxidative stress, and **(Q)** constitution of the mucosal barrier. qPCR data are represented with scatter plot and boxplot overlays (n = 8-9 per injection/infection group). The scatter plot differentiates the additional biological replicates included in the qPCR validation (solid dots) from those selected for microarray analysis (empty dots). Lowercase letters indicate significant differences between groups, as determined by estimated marginal means. **(R)** Summary of the results from the generalized linear model (GLM) analysis of the qPCR data, and the Pearson correlation analysis of gene transcript levels and total lice counts. Upward and downward arrows indicate significant up and down-regulation, respectively. Asterisks are used instead of arrows when significant lice and ASAL interaction (i.e., lice*ASAL) was detected. Asterisks also indicate significant Pearson correlation. The statistical significance threshold was *p <* 0.05 for all statistical analyses. *emc10*’s, *calr3a*’s, and *anxa4*’s Pearson coefficients are indicated due to their closeness to statistical significance (i.e., 0.05 < *p* < 0.10) and physiological relevance.

Among the GOIs putatively involved in cell adhesion, wound healing and mucosal barrier constitution, *vascular cell adhesion molecule 1 B* (*vcam1b*), *matrix metallopeptidase 2 A* (*mmp2a*), *cathepsin B* (*ctsb*), *ER membrane protein complex subunit 10* (*emc10*), *calreticulin 3 A and B* (*calr3a, calr3b*)*, annexin A4* (*anxa4*), and *mucin 2* (*muc2*) were up-regulated by lice (GLM results; **Figure 8R**). Lice-infected salmon groups showed higher *mmp2a* and *muc2* mRNA levels than the non-infected (**Figures 8D, Q**). For *vcam1b* and *anxa4*, ASAL/lice salmon showed higher transcript levels than the non-infected salmon (**Figures 8B, L**). ASAL showed a trend (i.e., close to statistical significance; p = 0.07; **Figure 8R**) towards *ctsb* down-regulation, which resulted in significantly lower mRNA levels in ASAL/no lice salmon than lice-infected salmon (**Figure 8H**). The same pairwise differences were found for *calr3a* (**Figure 8J**), and similar pairwise differences for *emc10* (i.e., non-infected < PBS/lice; **Figure 8I**) and *calr3b* (i.e., ASAL/no lice < PBS/lice; **Figure 8K**); however, no ASAL effects were detected by the GLM analysis for these genes (**Figure 8R**). The transcript levels of *matrix metallopeptidase 14* (*mmp14*) and *plasminogen activator inhibitor 1* (*serpine1*) showed an overall induction by ASAL (**Figure 8R**). ASAL/lice salmon had higher mmp14 expression levels than PBS-injected salmon (**Figure 8G**). serpine1 did not present significant pairwise differences between groups (**Figure 8M**). As a result of ASAL*lice interaction, lice infection up-regulated *glucosamine (UDP-N-acetyl)-2-epimerase/N-acetylmannosamine kinase* (*gne*) only in the ASAL-injected salmon (i.e., ASAL/lice > ASAL/no lice; **Figures 8C, R**), and down-regulated *mmp13a* only in the PBS-injected salmon (i.e., PBS/lice < PBS/no lice; **Figures 8E, R**), and PBS/lice salmon showed higher *sesn1a* mRNA levels than the rest of the injection/infection groups (**Figures 8P, R**). The GOIs *vascular cell adhesion molecule 1 A* (*vcam1a*), *matrix metallopeptidase 13 B (mmp13b)*, *actinin alpha 1* (*actn1*), and *heme oxygenase 1* (*hmox1*) did not show significant ASAL or lice effects. Total lice load counts were significantly negatively correlated with *ctsb, calr3b*, and *actn1* (**Figure 8R**).

### Identification of Gene Expression Patterns

**Figure 9:**
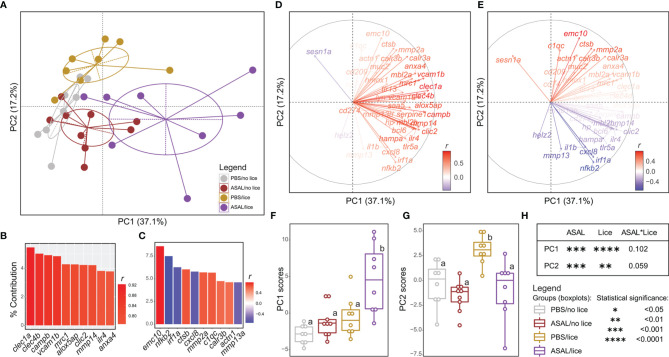
Principal component analysis (PCA) of the microarray validation qPCR data. **(A)** PCA plot illustrating the distribution of all salmon included in the qPCR validation experiment (n = 33) in the multivariate space. The dots on the plot represent salmon and are colored based on the injection/infection group to which they belong. **(B, C)** Bar plot representing the top 10 GOIs contributing (%) to the PC1 and PC2 variances, respectively. Each GOI’s bar is colored based on their correlation with the variance explained by PC1 **(B)** and PC2 **(C)**. **(D, E)** Loading vector plot showing the association of each GOI with the variance explained by PC1 and PC2. Vectors are colored based on their correlation with PC1’s **(D)** and PC2’s **(E)** variances. **(F, G)** Scatter plot/boxplot overlays representing individual/group PC1 and PC2 score data, respectively. Lowercase letters indicate significant differences between groups, as determined by estimated marginal means. **(H)** Summary of the results from the generalized linear model (GLM) analysis of the PC1 and PC2 score data. The significance threshold was *p < *0.05 for all statistical analyses.

The first two principal components of the PCA explained 54.3% of the variance in the qPCR-analyzed transcripts’ RQs and separated the treatment groups in the multivariate space (**Figure 9A**). Principal component 1 (PC1) segregated the ASAL/lice salmon (right) from the PBS/lice and ASAL/no lice salmon (center), and the PBS/no lice (left). Principal component 2 (PC2) segregated the PBS/lice salmon (top) from the other three groups (bottom). The top 10 transcripts contributing to PC1 comprised namely transcripts up-regulated by lice infection and ASAL injection in an additive (e.g., *clec1a*, *clec4b*, *mrc1*) or synergistic (e.g., *alox5ap*, *clic2*, *il4r*) fashion (**Figure 9B**). All transcripts except for *sesn1a* (lice-induced only in PBS-injected salmon) and *helz2* (lice-repressed) had positive PC1 loadings (**Figure 9D**). Hence, ASAL/lice salmon presented the highest PC1 scores. On the other hand, the top 10 transcripts contributing to PC2 were either up-regulated by lice (positively correlated with PC2 scores; e.g., *emc10*, *ctsb*, *mmp2a*) or ASAL (negatively correlated with PC2 scores; e.g., *nfkb2*, *irf1a*, *cxcl8*) (**Figure 9C**). Consequently, PBS/lice salmon showed the highest PC2 scores, and ASAL/no lice the lowest. As shown in **Figures 9F–H**, ASAL and lice-derived effects on PC1 and PC2 scores were statistically significant.

### Complementary qPCR Experiment (Att *vs*. Adj Skin Sites)

**Figure 10:**
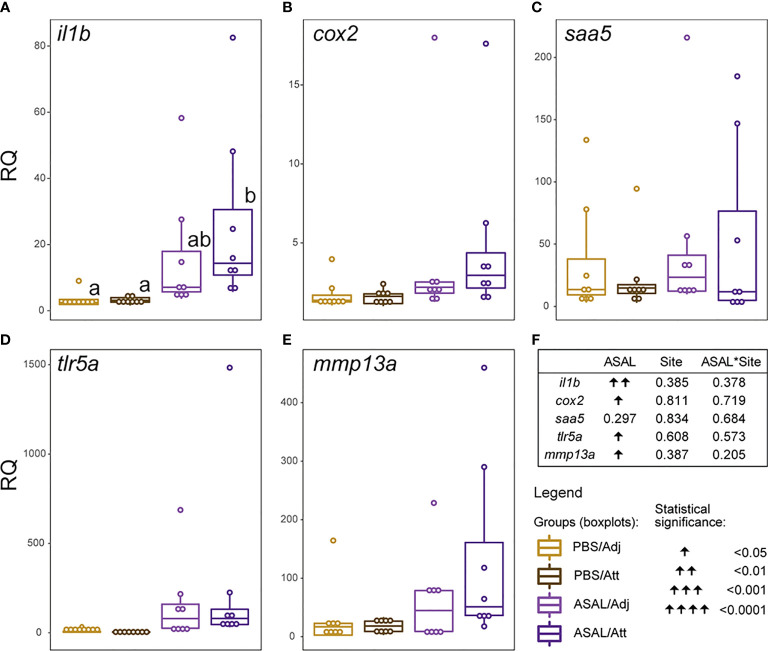
**(A–E)** Scatter plot/boxplot overlays representing individual/group data of the complementary qPCR experiment examining the differences in the expression levels of a selection of immune-relevant biomarker genes between louse-attachment (Att) and adjacent (Adj) skin sites. Lowercase letters indicate significant differences between groups, as determined by estimated marginal means. **(F)** Summary of the results from the generalized linear model (GLM) analysis of the qPCR data. The significance threshold was *p < *0.05 for all statistical analyses.

None of the selected transcripts showed significantly different expression levels between louse attachment (Att) and adjacent skin sites (Adj) (**Figures 10A–F**). All transcripts except for *saa5* (**Figure 10C**) were significantly up-regulated by ASAL.

## Discussion

### Skin Transcriptome Response to Lice Infection and Its Modulation by the ASAL Bacterin Stimulus

Pre-adult *L. salmonis* infection provoked extensive transcriptomic changes (i.e., 2,534 DEPs; **Figure 2**) in the skin of the PBS-injected salmon [i.e., Lice(PBS) list], characterized by the predominance of repressed transcripts putatively involved in housekeeping metabolic and cellular processes such as nucleic acid/protein metabolism (**Figure 6**). Umasuthan et al. (34) reported similar biological processes as dysregulated by *L. salmonis* chalimus I in the fins of Atlantic salmon, although some of these processes were represented mainly by induced genes. The overwhelming over-representation of housekeeping biological processes highlights the lesser representation of transcripts with putative roles in immune/stress processes. Furthermore, most immune/stress response-related transcripts were repressed as well. As confirmed by qPCR analysis, lice infection did not induce the inflammation biomarker genes *il1b*, *cxcl8*, and *cox2* (52–55) at the louse attachment and/or adjacent dorsal skin sites of PBS-injected salmon (**Figures 7**, **10**). The transcription of *il1b* and *cxcl8* had not shown a significant response to pre-adult *L. salmonis* infection in Atlantic salmon intact dorsal skin (56), nor had they been microarray-detected in *L. salmonis-*damaged skin compared with intact skin (24). Sea lice infection has repeatedly been described as immunosuppressing Atlantic salmon directly –*via* secretion of inflammation response-inhibitory substances– and indirectly –*via* chronic stress effects– (9, 11, 57). Chronic stress can also force fish into adaptive physiological changes (58–60), which could be responsible for the aforementioned repression of metabolic and cellular processes. Also, Atlantic salmon have shown opposed transcriptomic responses depending on the sea lice life stage (11), which may explain the discrepancy between the present study and Umasuthan et al. (34). Further research is required to determine whether such repression response reflects a physiological coping mechanism by the salmon upon lice infection or the salmon’s metabolism reprogramming by the parasite.

The present microarray and qPCR analyses evidenced pre-adult *L. salmonis* immunosuppressive effects on the transcript levels of interferon (IFN)-stimulated genes in the PBS-injected salmon (**Figure 7** and **Supplemental Table S2**); for example, *radical S-adenosyl methionine domain-containing 2* (*rsad2*, alias *viperin*) (46, 61, 62), *interferon-induced protein with tetratricopeptide repeats 5* (*ifit5*) (46, 63, 64), or *helz2* (46, 65). In fact, 6 of the GO terms over-represented in Lice(PBS) referred to antiviral processes (e.g., “defense response to virus”). The available literature demonstrates that *L. salmonis* parasitism hinders Atlantic salmon antiviral responses, thus increasing their susceptibility to viral infection [e.g., to infectious salmon anemia virus (ISAv)] (11, 66). In addition to IFN-dependent signaling pathways, *L. salmonis* infection in the PBS-injected salmon repressed many genes involved in mitogen-activated protein kinase (MAPK) pathways [e.g., *TNF receptor-associated factor 2* (*traf2*), *mapk8*, *interleukin-1 receptor-associated kinase 4* (*irak4*)] and stress response [e.g., several heat shock protein (HSP)-encoding genes].

Despite the absence of a clear pro-inflammatory gene expression profile, the Lice(PBS) list presented several up-regulated transcripts putatively involved in fish skin immune defense against pathogens (**Figure 7** and **Supplemental Table S2**). For example, the increased expression of *c1qc* and *mbl2a* suggests the activation of both classical and lectin complement systems, respectively (67, 68). The qPCR analyses also confirmed the slight up-regulation of *cd74* by *L. salmonis* infection, which may suggest enhanced MHCII molecule transport for endocytic antigen capture (69). Increased mucus production is a typical feature in Atlantic salmon skin’s response to *L. salmonis* infection (11). In agreement with the latter, *muc2* –encoding a constituent of fish skin mucus (70)– was one of the most intensely induced genes in the Lice(PBS) list. Also, some dysregulated metabolic or cellular processes in Lice(PBS) (e.g., “protein modification process”) can be linked to stress response through the up-regulated genes representing them [e.g., *hypoxia-inducible factor 1-alpha* (*hif1a*), a hypoxia biomarker in fish (71); *mapk8*, reported as responsive to hypotonic stress in *Lateolabrax maculatus* (72); *sesn1a*, involved in cell protection against oxidative stress (73)].

The Lice(PBS) list was also characterized for the lack of over-represented development/healing-related biological processes (**Figure 3**). The list, nevertheless, included some up-regulated genes putatively related to wound healing (**Figure 8** and **Supplemental Table S2**): e.g., *anxa4* [tissue regeneration (74)], *calr3a* and *calr3b* [hypertrophy-like and thrombopoiesis processes (75, 76)], *periostin* [*postn*; ketatinocyte proliferation, myofibroblast differentiation, and fibrillogenesis (77, 78)], and *mmp2a* [scarring resolution (79)]. In the present study, *mmp2a* was the only microarray-detected matrix metalloproteinase-encoding gene up-regulated by *L. salmonis* infection in PBS-injected salmon –*mmp13a* was lice-repressed–. Umasuthan et al. (34) found decreased transcript levels of *mmp2* in Atlantic salmon fins infected with chalimus *L. salmonis*. Discrepancies in *mmp* (e.g., *mmp13*, *mmp9*, *mmp2*) transcriptional patterns are common in *L. salmonis* infection research (24, 27, 28, 34, 80), especially when different sea lice life stages are considered. Like Skugor et al. (24), the protease-encoding transcript *ctsb* was lice-induced in the Atlantic salmon dorsal skin. ECM degradation by CTSB enables the recruitment of keratinocytes in the wound area in mammals (81). The up-regulation of *emc10* by lice infection supports the endothelial cell migration promotion hypothesis (i.e., *mmp2a* up-regulation) and may suggest new blood vessel formation in the PBS-injected fish (i.e., angiogenesis), based on the mammalian literature (82). Angiogenesis is a necessary process during the proliferative phase of wound healing (78). Some parasites are known to stimulate angiogenesis in their human (83) and fish (84) hosts. However, it seems unlikely that *ctsb* and *emc10* up-regulation could favor *L. salmonis* infection since *ctsb* was negatively correlated with total lice counts, while *emc10* showed a similar trend (i.e., almost significantly correlated; *p* = 0.06). Alternatively, increasing the skin’s microvessel density could be a protective mechanism to improve the recruitment of cells with anti-parasitic and wound healing roles (85). In sum, the above results may suggest the occurrence of molecular changes in the intact skin to support wound healing at the louse attachment sites.

Compared with the PBS-injected salmon, lice infection had a substantially lesser effect on the skin transcriptome in the ASAL-injected salmon [i.e., 85 DEPs in the Lice(ASAL) list *vs.* 2,534 in the Lice(PBS) list; **Figure 2**]. Furthermore, in contrast with Lice(PBS), Lice(ASAL) was mostly composed of lice-induced transcripts putatively involved in immune/stress and development/healing-related processes (**Figure 6**). Additionally, the microarray results suggest ASAL injection mitigated lice repressive effects on these genes [i.e., not detected in the Lice(ASAL) list]. As evidenced by ASAL(lice) lists, the ASAL bacterin injection induced a strong immune response in the lice-infected salmon skins (discussed in the next section).

The ASAL-injected salmon showed signs of a more robust immune response to *L. salmonis* infection than the PBS-injected fish. The Lice(ASAL) list presented highly lice-induced genes (i.e., fold-change >2; **Figure 7** and **Supplemental Table S2**) with roles in APRs [i.e., *saa5* (86)], eicosanoid synthesis [i.e., *arachidonate lipoxygenase 3* (*aloxe3*) (87)], antiviral responses [e.g., *interferon-induced protein 44* (*ifi44*) (62, 65)], and T helper 2 (Th2)-type immune response [i.e., *interleukin-13 receptor subunit alpha-2* (*il13ra2*) (88)]. Besides, the Lice(ASAL) list over-represented biological processes related to neutrophil degranulation (**Figure 3**), which is a key process in innate immune responses (89). Moreover, a pronounced influx of neutrophils in the inflammation site seems to be one of the main features of lice-resistant coho salmon (7, 9, 90). In this sense, the microarray analysis also identified several lice-induced genes potentially encoding neutrophil granule proteins (91) such as antimicrobial peptides (e.g., *camp*), proteases [*ctsb, mmp2a*, *disintegrin and metalloproteinase domain-containing protein 9* (*adam9*)], and *heat shock 70 kDa protein 4L* (*hspa4l*). Like the PBS-injected salmon, *cxcl8* –which encodes a known neutrophil chemoattractant (88, 92)– was not significantly lice-induced in the ASAL-injected salmon. Yet, *cxcl8* was one of the main contributors to the segregation of PBS/lice salmon (i.e., no-inflammatory-response phenotype) from the other groups in the PCA (**Figure 9**). Also, as Braden et al. (90) argued, the induction of acute-phase protein (APP)-encoding genes in the skin –*saa5* in the present study– could be behind the recruitment of inflammatory cells in lice-infected Atlantic salmon. Another finding suggesting anti-lice properties for the ASAL treatment was the up-regulation of *hampa*, which was only observed in the ASAL-stimulated salmon. HAMP decreases the availability of iron in plasma (93), thus constituting an effective defense mechanism against hematophagous parasites like *L. salmonis*. Indeed, lice-resistant salmonid species and Atlantic salmon fed an anti-lice functional diet showed a strong induction of genes encoding iron-binding proteins (26, 94). Finally, Th2-type gene expression signatures have also been found in the skin of *L. salmonis-*resistant salmonid species (90). The lice-induction of *il13ra2*, which encodes an IL13 decoy receptor (88), could be interpreted as a sign of Th2 polarization inhibition. However, the concomitant up-regulation of *il4r* [Th2 cell biomarker (88)] and genes involved in wound healing (discussed below) may suggest the contrary.

The much smaller size of the Lice(ASAL) list compared with Lice(PBS) may be due to the ceasing of the lice-repressing effects on genes involved in cell housekeeping processes. The dissolution of such extensive transcriptomic changes within the ASAL-injected group made skin development-related transcripts proportionally more important in the Lice(ASAL) list. In addition, 3 of these genes [i.e., *aloxe3*, *desmocollin-2* (*dsc2*), and *fibroblast growth factor receptor 1* (*fgfr1*)] had induction fold-changes amply above (e.g., 15-fold up-regulation for *aloxe3*) those of any of the lice-induced genes in the Lice(PBS) list (**Supplemental Table S2**). ALOXE3 participates in forming the skin permeability barrier in humans (86) and was induced upon thermal stress in the Antarctic fish *Notothenia coriiceps* (95). DSC2 is a desmosomal cadherin that mediates in mammalian and fish tissue development processes involving cell-cell adhesion (96) and has been found up-regulated in human venous ulcers (97). FGFR1 knockout in murine keratinocytes impaired their migration at the wound edge (98). Other up-regulated tissue regeneration-relevant genes in Lice(ASAL) may imply the stimulation of the canonical Wnt pathway and epithelial-mesenchymal transition [i.e., *catenin beta-1* (*ctnnb1*) (99)], cornification [i.e., *keratin 8* (*krt8*) and *envoplakin* (*evpl*) (100)], and cell-cell and cell-ECM adhesions [i.e., *integrin subunit alpha V* (*itgav*) (101), *fibrillin-1* (*fbn1*) (102), *adam9* (103), and *ctsb* (81)]. In sum, as a transient activator of the skin’s immune defenses, it could be hypothesized that ASAL injection could have mitigated some of the adverse physiological effects of *L. salmonis* infection [e.g., immunosuppression, impaired wound healing (11)].

### Skin Transcriptome Response to ASAL Bacterin and Its Modulation by Lice Infection

The present study provides the first insights into Atlantic salmon’s skin transcriptomic response 24 h after an intraperitoneal injection of an *A. salmonicida* bacterin vaccine (ASAL) and its modulation by *L. salmonis* parasitism. Similar ASAL preparations elicited strong anti-bacterial gene expression responses in the spleen and head kidney of IP-treated steelhead trout (*Oncorhynchus mykiss*) (104) and Atlantic cod (29–31). Herein, the transcriptome of Atlantic salmon’s skin showed significant changes in response to ASAL (**Figure 2**), mostly comprised of up-regulated transcripts putatively related to immune/stress processes (**Figure 6**). The magnitude of these responses was markedly influenced by the absence/presence of *L. salmonis* infection, with the lice-infected showing a larger number of DEPs than the non-infected [i.e., 283 DEPs in the ASAL(lice) *vs.* 27 in the ASAL(no lice)]. Nevertheless, and in alignment with previous studies on fish systemic response to ASAL (29–31, 104), both lists shared up-regulated genes encoding proteins putatively involved in iron homeostasis [i.e., *hampa* (93)], inflammation [e.g., *cd274* (alias *pdl1*), an M1 macrophage biomarker (105)], and proteolysis-mediated immune processes [i.e., *cathepsin L* (*ctsl*), with roles in apoptosis, ECM degradation, antigen processing, and mucosal immunity (106–108)] (**Figure 7** and **Supplemental Table S2**). In general, *L. salmonis*-infected and non-infected salmon shared gene expression signatures suggesting enhanced leukocyte recruitment [i.e., *C-C motif chemokine 2* (*ccl2*, alias *mcp-1*), *lyn*, and *clic2* (109–111)] and infiltration [i.e., *high affinity immunoglobulin gamma Fc receptor I* (*fcgr1a*) (112)], possibly aided by increased angiogenesis and vessel permeability [i.e., *G-protein coupled receptor 4* (*gpr4*) (112, 113)].

The larger number of ASAL-responsive DEPs in the skin of sea lice-infected salmon [i.e., ASAL(lice)] may signify a more vigorous response to the bacterin and the overcoming of some of the lice immunosuppressive effects discussed above. The microarray data from the PBS/lice salmon suggested changes in the skin’s cell composition (e.g., enrichment in keratinocytes and fibroblasts) and increased angiogenesis, which could be a contributing factor to the higher magnitude of the ASAL response in the lice-infected salmon. The limited number of GO terms over-represented in ASAL(no lice) prevented identifying sea lice modulatory effects on the Atlantic salmon skin’s response to ASAL at the biological process level. In any case, the list of over-represented biological processes in ASAL(lice) provided a well-defined picture of the anti-bacterial skin transcriptome response to ASAL in the *L. salmonis*-infected salmon (**Figure 4**).

ASAL response in *L. salmonis*-infected salmon dysregulated molecular pathways related to PAMP detection by pathogen recognition receptors (PRRs; **Figure 4**). Contributing to the over-represented GO term “toll-like receptor signaling pathway”, there were genes encoding proteins involved in NF-κB activation *via* myeloid differentiation primary-response protein 88 (MyD88)-dependent signaling [*irak4* (114), *baculoviral IAP repeat-containing protein 3* (*birc3*, alias *ciap2*) (115)] and TRIF-dependent [*TRAF family member-associated NF-kappa-B activator* (*tank*) (116)] toll-like receptor (TLR) cascades (**Supplemental Table S5**). The over-representation of the terms “response to lipopolysaccharide” and “defense response to Gram-positive bacterium” suggests that the detection of different *A. salmonicida* PAMPs [e.g., lipopolysaccharides (LPS), peptidoglycans] contributed to the observed transcriptomic response to ASAL. The qPCR analysis found two PRR-encoding transcripts *tlr5a* [bacterial flagellin detection (117)] and *tlr13* [bacterial 23S rRNA detection (118)] induced in ASAL/lice compared with PBS/lice salmon (**Figure 7**). The ASAL induction of *TRAF-interacting protein with FHA domain-containing protein A* (*tifa*) and *C-type lectin domain family 4 member D* (*clec4d*) may imply the dysregulation of two additional PRR pathways: the α-kinase 1 (ALPK1)-TIFA signaling pathway (119); and the C-type lectin receptor (CLR)/tyrosine-protein kinase (SYK) signaling pathway (120) (**Supplemental Table S2**).

NF-κB and MAPK pathways activation after PAMP detection induce the production of pro-inflammatory cytokines by innate immune cells (114, 117, 121), such as TNFA, IL1B, IL6, and IL18; and pro-inflammatory prostaglandins *via* increased COX2 expression. ASAL-injected lice-infected salmon showed up-regulated mRNA levels of *il1b* and *cox2* (**Figure 10**) and *il18* (**Supplemental Table S2**), as well as multiple genes over-representing “response to interleukin-1” and “response to interleukin-6” (**Supplemental Table S5**). Previous studies of ASAL-challenged fish showed *tnfa* and *il1b* mRNA levels in the spleen and head kidney decreased rapidly after peaking at 3-6 h post-exposure (31, 104). Considering that the skin samples were collected 24 h post-PBS/ASAL treatment, the slight *il1b* and absence of *tnfa* and *il6* induction observed here may reflect the normal progression of the molecular response of Atlantic salmon skin to ASAL. Nevertheless, as discussed below, a cascade of molecular events triggered by these pro-inflammatory cytokines could be inferred based on the ASAL(lice) list.

IL1B, IL6, and lipid mediators synthesized by the COX2 pathway alter the surrounding cells’ and tissues’ function and structure and cause increased vascular permeability, swelling, cell adhesion, and angiogenesis while promoting the activation, proliferation, and differentiation of leukocytes in mammals and fish (52, 85, 122). The ASAL(lice) list suggests that ASAL treatment in *L. salmonis*-infected salmon activated pathways promoting angiogenesis and endothelial barrier permeability [e.g., *G-protein coupled receptor 4* (*gpr4*) up-regulation (112, 113)], and cell adhesion [i.e., the induction of *intercellular adhesion molecule 1* (*icam1*) and *E-selectin* (*sele*) (85, 113, 123)] (**Supplemental Table S2**). The ASAL(lice) list also showed a slight up-regulation of *serpine1* (**Figure 8**), which is involved in blood coagulation (hemostasis), and cell adhesion and migration (124, 125). *Flavobacterium columnare* infection induced *serpine1* expression in the skin of channel catfish (*Ictalurus punctatus*) (126). ECM degradation by MMPs and other proteinases enables angiogenesis and cell migration –hence, it facilitates leukocyte recruitment at the infection/inflammation site (85, 127). Here, ASAL up-regulated *mmp14* in the skin of lice-infected salmon (**Figure 8**). Zebrafish MMP14 has been determined as collagenolytic and necessary for scar resolution (128). The intestine of Japanese flounder (*Paralichthys olivaceus*) showed high *mmp14* induction after immersion vaccination with live attenuated *Edwardsiella tarda*, which was proposed as a means of enhancing cell migration (129). Taken together, the aforementioned ASAL-induced genes depict putative changes in the skin microvasculature and endothelial function consistent with inflammation and increased leukocyte recruitment.

Within the *L. salmonis*-infected salmon, and besides *serpine1*, the ASAL treatment activated the transcription of other genes involved in hemostasis (**Supplemental Table S2**) such as *fibrinogen alpha chain* (*fga*) and *P2Y purinoceptor 1* (*p2ry1*). Fibrinogen is cleaved by thrombin into fibrin, a major component of blood clots, which also assists in tissue repair and immune processes by accumulating phagocytes, endothelial cells, and fibroblasts, as well as cytokines and growth factors (130, 131). As for *p2ry1*, mammalian P2RY1 (alias P2Y1) is known to exert pro-coagulant effects by mediating in platelet aggregation (132). On the other hand, the ASAL(lice) list also revealed increased mRNA levels of *thrombomodulin* (*thbd*), a gene known in mammals for the anti-coagulant function of its protein product (133). ASAL also up-regulated *coagulation factor V* (*f5*) in the lice-infected salmon. Post-translational modifications can confer the mammalian F5 either pro- or anticoagulant activity (134). All in all, these results suggest that the coagulation cascade was activated by *A. salmonicida* bacterin in the intact skin of lice-infected salmon, agreeing with previous studies on fish mucosal tissues exposed to live bacterial pathogens and antigens (126, 129, 135).

Several of the hemostasis-relevant proteins discussed above (e.g., SERPINE1, FGA) fall within the category of APPs in many vertebrates, including fish (136). Although APPs are predominantly expressed by hepatocytes to be secreted into the blood, they can also be produced by endothelial cells and leukocytes activated by pro-inflammatory cytokines (e.g., IL1B and IL6) at the site of infection (136, 137). The ASAL(lice) list showed induced *saa5* transcript levels in the lice-infected salmon (**Figure 7** and **Supplemental Table S2**). SAAs are some of the best-known APPs in vertebrates and play various roles in APRs (e.g., lipid metabolism regulation, immunomodulatory activity) (136, 137). The ASAL induction of *hampa* and, at a much lower extent, *hp* may have been intended to reduce iron availability for bacterial growth (93, 138). Proteins and transcripts in the complement system –often referred to as involved in APRs (136)– have been identified as responsive to bacterial infection in the fish skin mucus (139). The ASAL(lice) list showed up-regulated transcript levels of *mbl2b*, *complement component C7* (*c7*), and *C3a anaphylatoxin chemotactic receptor* (*c3ar1*). Interestingly, *mbl2a* only responded to *L. salmonis* infection, which may suggest regulation divergence between the two *mbl2* paralogues and, possibly, different functions [e.g., complement pathway activation after binding lice (MBL2A) or *A. salmonicida*-specific (MBL2B) carbohydrate PAMPs]. In mammals, C7 takes part in the lysis of target pathogen’s membranes as a component of membrane attack complex (MAC) (68, 140), whereas C3 promotes chemotaxis, degranulation, and reactive oxygen species (ROS) production in C3AR1-expressing myeloid cells (e.g., granulocytes, macrophages) (68). Outside the complement system, ASAL also up-regulated the transcription of *lysozyme C* (*lyz*) and *campb* within the *L. salmonis*-infected salmon. Fish LYZ and CAMP functional characterization has evidenced bacterial cell wall-lysing activity (141, 142), and both proteins are part of fish skin bactericidal weaponry (70, 139, 143).

In sum, the ASAL-injected Atlantic salmon’s skin transcriptome showed traits of M1/Th1 (i.e., cytotoxic) or M2 (macrophage)/Th2 (i.e., tissue repair)-type immune responses. The activation of M1/Th1 marker genes by ASAL [e.g., *ccl2, cd274*, *cxcl11*, *cxcl8*, *cxcl11* (88, 105, 144–146)] was unequivocal (**Figure 7** and **Supplemental Table S2**). However, the present microarray analyses also revealed the putative activation of molecular countermeasures to keep skin’s inflammation and cytotoxic responses to ASAL in check, as inferred from the increased mRNA levels of *tank*, *interleukin-1 receptor type 2* (*il1r2*), *pyrin* (*mefv*), and *guanylate-binding protein 1* (*gbp1*). The anti-inflammatory mechanisms represented by these genes involve the inhibition of 1) pro-inflammatory cytokine production [for *tank* (116)], 2) IL1B signaling [for *il1r2* (147) and *mefv* (148)], and 3) pro-inflammatory feedback loops [for *gbp1* (149)]. The fish skin was previously described as naturally skewed towards the Th2 phenotype (150), possibly for protection against ectoparasites and inflammation-derived self-damage.

### Atlantic Salmon Skin Transcriptome Response to Lice and ASAL Co-Stimulation

The discussion of the *L. salmonis* infection and ASAL injection co-stimulated genes (i.e., COS list, corresponding to the ASAL/lice *vs*. PBS/no lice comparison) is tightly interwoven with that of the single-stimulus DEP lists, given their large proportion of overlapped DEPs (**Figure 2**).

Lice(PBS) and COS lists were enriched with genes involved in basic housekeeping metabolic and cellular processes such as gene expression regulation and organelle biogenesis (**Figure 5**). However, the ratio of up-regulated/down-regulated genes was more balanced in the COS over-represented metabolic and cellular processes than those corresponding to the Lice(PBS) list. As also suggested by the Lice(ASAL) list, the ASAL treatment appeared to have partially mitigated *L. salmonis* repressive effects on these biological processes. Short-term acute stress challenges (e.g., *A. salmonicida* bacterin treatment) can revert the detrimental physiological effects of long-term chronic stress (e.g., lice infection) (60).

Lice infection alone did not affect the transcript levels of the pro-inflammatory cytokines *il1b*, *ccl2*, *cxcl8*, and *il18* (52, 53, 109); however, lice-infected salmon showed a stronger ASAL induction of these genes compared with the non-infected salmon [i.e., significant differences in ASAL(lice), not in ASAL(no lice)] (**Figure 7** and **Supplemental Table S2**). The COS list also identified significantly increased expression levels for these cytokine-encoding genes; however, their COS fold-changes were relatively lower than those of the ASAL(lice) list. The higher ASAL-induction in ASAL(lice) *vs*. COS lies in the fact that the first list emphasized ASAL effects over those of lice [i.e., PBS/lice as the reference group in ASAL(lice)]. This phenomenon does not apply to the COS list, which still captures some of the deleterious effects of the parasites on the skin’s physiology (i.e., PBS/no lice fish as the reference group).

The COS list conserves the lice-elicited down-regulation of key antiviral IFN-stimulated genes [e.g., *rsad2*, *ifit5*, *helz2* (46, 61–65)] detected in the Lice(PBS) list (**Figure 7** and **Supplemental Table S2**). Based on ASAL(lice) and ASAL(no lice) lists, ASAL stimulus did not seem to affect the transcription of these antiviral genes. This lack of responsiveness to bacterial stimuli was previously reported for *rsad2* in the Atlantic cod spleen and leukocytes challenged with formalin-killed *A. salmonicida* and LPS (29, 151). Nevertheless, in contrast to Lice(PBS), the COS list over-represented biological processes related to the activation of innate immune response and myeloid cells (e.g., neutrophils).

Similar to Lice(ASAL) –but to a much greater extent–, the COS list included tens of up-regulated genes encoding proteins found in human neutrophil granules (91) (**Figure 7** and **Supplemental Table S2**). That is the case of the antimicrobial peptides CAMPA and LYZ [cell wall-lysis (141, 142)], the proteinases MMP2A and MMP14 [tissue remodeling and repair *via* ECM degradation (81, 128)], and the hemoglobin-binding protein HP [bacterial growth hampering (138)]. The COS list presented genes for additional neutrophil granule proteins involved in tissue remodeling and wound healing [e.g., *ctsl*, *ctsz* (107)], cell process protection against stress [e.g., *hsps* (152)], and adaptive immune responses such as T cell activation and differentiation [e.g., *plastin-2* (*lcp1*), *fcer1g* (153, 154)]. The COS list also evidenced the co-stimulation of *integrin beta-3* (*itgb3*) transcript levels. ITGB3 was previously found in human neutrophil granules (91) and was described to participate in blood coagulation *via* platelet activation (155).

The neutrophil-related genes in the COS list support the notion that ASAL+lice may have activated key wound healing, angiogenesis, and hemostasis processes –as discussed above for Lice(ASAL) and ASAL(lice) lists. Further, 3 GO terms related to endothelial cell migration –a process only hinted at as lice-activated by *mmp2a* and *emc10* in Lice(PBS) (as previously discussed)– were over-represented by 48-89 DEGs in the COS list (**Figure 5** and **Supplemental Table S7**). Among these COS DEGs, there were up-regulated genes with putative roles in endothelial cell migration promotion [e.g., *itgb3, fgfr1*, *tumor necrosis factor receptor superfamily member 5* (*cd40*) (156–159)], and down-regulated genes putatively involved in endothelial cell migration inhibition [for example, various *histone deacetylases* (e.g., *hdac2*, *hdac7*) (160)]. Furthermore, the COS list over-represented “angiogenesis”, which included DEGs shared with ASAL(lice) and ASAL(no lice) lice [e.g., *gpr4* (112, 113)], but also COS-exclusive DEGs [e.g., *vascular endothelial growth factor receptor 2* (*kdr*) (157)]. Inflammatory and healing processes are enabled by the binding of migrating cells (e.g., leukocytes, platelets) to the damaged or infected tissue (85, 161). Similar to ASAL(lice), the COS list presented several up-regulated genes encoding adhesion molecules for cell-ECM [e.g., *itgav* (101)], leukocyte-endothelium [e.g., *icam1*, *vcam1b* (123)].

As seen for ASAL within the lice-infected salmon [i.e., ASAL(lice)], ASAL+lice co-stimulation (i.e., COS) increased the mRNA levels of several hemostasis-related genes [e.g., *serpine1*, *fga*, *p2ry1*, *itgb3* (124, 125, 130, 132, 155)] (**Supplemental Table S2**). Interestingly, several other DEGs in the COS list over-represented 2 biological processes related to platelet formation, which may suggest thrombocyte involvement in the skin response to ASAL+lice. This list of DEGs included the up-regulation of *tyrosine-protein phosphatase non-receptor type 6* (*ptpn6*) and *actn1*. Mammalian PTPN6 has been described to promote platelet activation through its mediation in different signaling pathways, including CLEC1B (alias CLEC-2), FCER1G, and ITGB3 (162). Upon binding to their ligands, platelet activation signaling continues its course *via* protein-tyrosine phosphorylation reactions catalyzed by tyrosine-protein kinases such as LYN (162). This molecular pathway is yet to be described in fish thrombocytes. However, the up-regulation of *fcer1g*, *clec1b*, *itgb3*, and *lyn* observed in the COS list and its potential thrombocyte connection deserves further investigation. Non-mammalian thrombocyte and mammalian platelet activation involves changes in their morphology that require the reorganization of the actin cytoskeleton (163). qPCR analyses could not confirm *actn1* co-stimulation, although it revealed its negative correlation with lice infection level. Although it is unclear how *actn1*’s unresponsiveness would have affected the hemostatic system of salmon, *L. salmonis* antithrombotic actions on Atlantic salmon have previously been reported (34).

The present analyses revealed ASAL+lice additive effects on the Atlantic salmon skin transcriptome, which resulted in over-representation of some of the COS-exclusive physiological features discussed above (e.g., platelet-like cell activation), and others involved in innate immune mechanisms. Regarding the complement system (68, 140), ASAL+lice co-stimulation induced *mbl2b*, *c3ar1*, *c3*, *c4*, *C4b-binding protein alpha chain* (*c4b*), and *c7* mRNA levels more intensely than single-stimulus exposures (**Figure 7** and **Supplemental Table S2**). The induction of several C-type lectin receptors was also strengthened by co-stimulation, thus alluding to pathways involved in platelet activation and dendritic cell motility (i.e., *clec1b*), as well as pathogen recognition and Th1/Th17 polarization [i.e., *C-type lectin domain family 4 member D* (*clec4d*, alias *mcl*) and *clec1a*] (120, 162, 164, 165). An anti-bacterial PRR-encoding gene, *tlr13* (118), also showed ASAL+lice additive interaction effects. One of the most highly up-regulated COS-exclusive genes was *troponin C* (*tnnc2*), which could be interpreted as an indication of a higher presence of pericyte-like cells (166). Since *tnnc2* was previously reported as *L. salmonis-*repressed in the skin of Atlantic salmon (27), its ASAL+lice co-stimulation provides additional evidence to ASAL enhancement of wound healing in lice-infected salmon. Like ASAL(lice) list, the COS list suggested lice infection boosted ASAL activation of antimicrobial (e.g., *hampa*, *campb*), chemotactic (e.g., *lect2*), and local acute phase (e.g., *saa5*) responses (53, 93, 136, 137, 141). Furthermore, some genes such as *hampa*, the Th2 cell biomarker *il4r* (88), and the M2 macrophage biomarker *alox5ap* (145) showed synergic (i.e., greater than additive) ASAL and *L. salmonis* interactive effects.

As suggested by the ASAL(lice) list and observed again in the COS list (**Figure 7** and **Supplemental Table S2**), the presence of increased mRNA levels of M1/Th1 (e.g., *cxcl8*, *lect2*, *clec4d*, *cd274*, *cxcl11*) and M2/Th2 (e.g., *il4r*, *ccr8*, *alox5ap*, *il1r2*) markers suggests a dual nature (i.e., anti-bacterial and tissue repair/protection) for Atlantic salmon’s skin response to ASAL+lice (88, 105, 144, 145, 164, 167). It could be argued that this intermediate phenotype could be beneficial for the fish skin since it would maintain its tissue repair capacity while fortifying its antimicrobial defenses.

Another important feature of the COS list is the exceptionally high presence (compared to the other lists) of genes involved in the adaptive immune system (**Figure 5**). ASAL+lice up-regulated the transcript levels of class I major histocompatibility complex (MHC) components and antigens [e.g., *beta-2-microglobulin* (*b2m*), *HLA class I histocompatibility antigens hla-a*], and components of immunoproteasomes [e.g., *proteasome subunit alpha type-6* (*psma6*), *beta type-5* (*psmb5*)] (88, 168). These DEGs collectively over-represented the GO term “antigen processing and presentation of peptide antigen *via* MHC class I”. At the same time, the COS list over-represented GO terms associated with negative regulation of B cell and immunoglobulin mediated processes (e.g., “negative regulation of B cell-mediated immunity”). The observed equilibrium in the ratio of up and down-regulated genes representing those processes suggests a fine-tuned control over antibody-mediated immune response to ASAL+lice co-stimulation. The present qPCR analyses confirmed the ASAL and *L. salmonis* synergic induction of *bcl6* (**Figure 7**), one of the better-characterized genes/proteins over-representing those GO terms. BCL6 is known in mammals as a transcriptional repressor of germinal center B cell differentiation into plasma cells while promoting B cell proliferation and the formation of high-affinity antibodies (169, 170). The development of vaccines has been and still represents a challenge in the immunization of farmed Atlantic salmon against sea lice (11). Given the present results, the potential use of bacterial PAMPs as sea lice vaccine adjuvant warrants further investigation.

**Figure 11:**
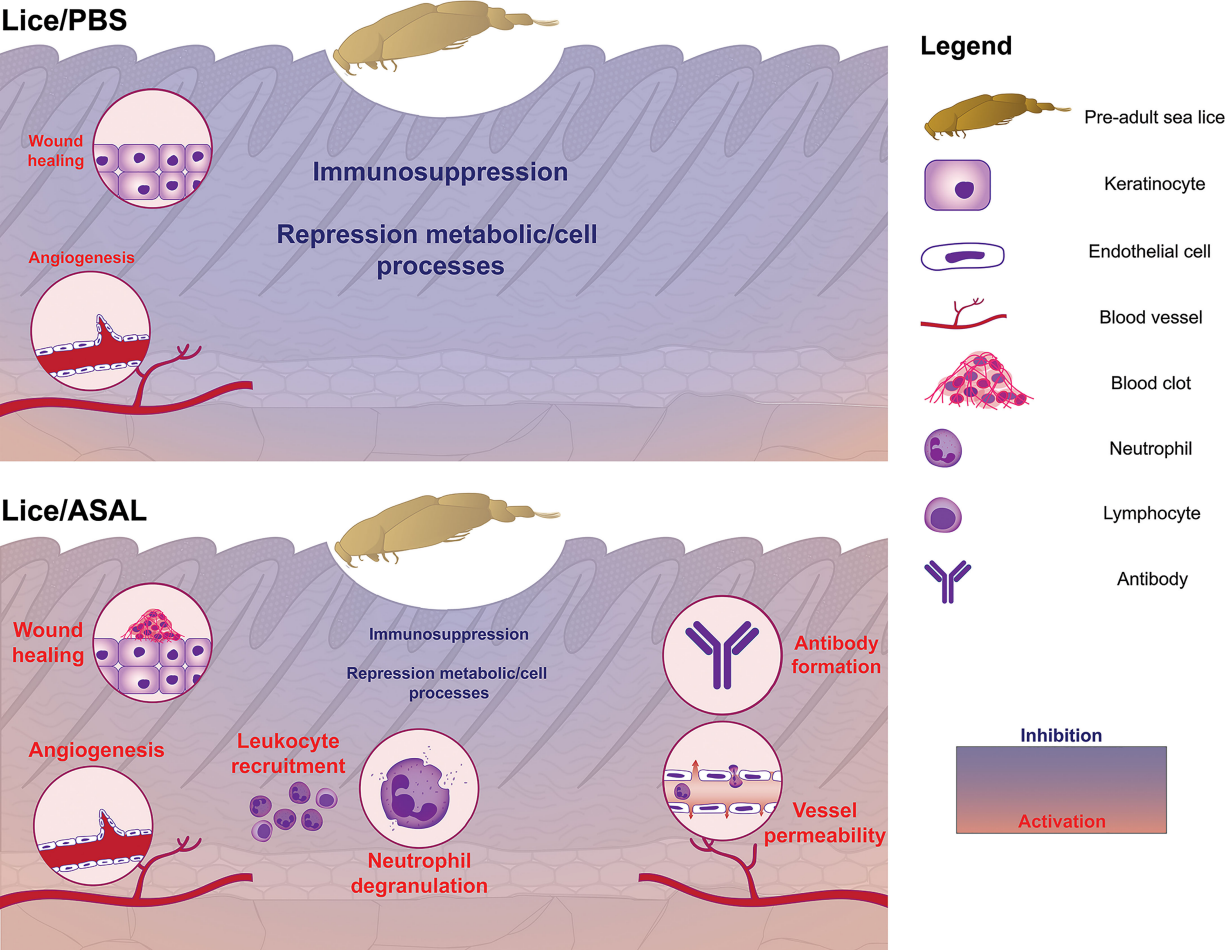
Summary of the main biological processes putatively regulated in response to sea lice infection in the skin of PBS-injected (Lice/PBS; top) and ASAL-injected (Lice/ASAL; bottom) Atlantic salmon, as explained in the Discussion. Biological processes’ names are colored based on whether they were inhibited (blue) or activated (red); their font sizes are proportional to their inhibition/activation extent. The stylized skin section used as the background was colored with a blue-red gradient color scheme representing the proportion of down-regulated/up-regulated genes in the Lice/PBS and Lice/ASAL salmon.

Overall, the COS list contributed to defining the ongoing physiological changes in the dorsal skin of the Lice/ASAL salmon compared with the Lice/PBS (**Figure 11**). First, ASAL attenuated –but did not resolve– some *L. salmonis* repressive effects on the transcript levels of antiviral biomarker genes and genes putatively involved in metabolic and cellular processes. Concomitantly, Lice/ASAL salmon showed increased transcriptomic changes suggesting increased leukocyte recruitment and the activation of innate (e.g., neutrophil degranulation) and adaptive (e.g., antibody formation) immune processes, which were not detected in the Lice/PBS salmon. Lastly, ASAL+lice co-stimulation also seemed to promote wound-healing (e.g., hemostasis) and developmental processes (e.g., angiogenesis).

## Concluding Remarks

The present study revealed significant interacting effects of *L. salmonis* and ASAL stimulation in the dorsal skin of Atlantic salmon. ASAL strengthened the immune gene expression response to *L. salmonis* infection (e.g., APR-and neutrophil degranulation-related genes) and mitigated lice repressive effects on fundamental cellular processes and some antiviral gene levels compared with the PBS controls [Lice(ASAL) *vs*. Lice(PBS)]. Vice versa, lice-infected salmon showed a more vigorous response to ASAL than the non-infected [ASAL(lice) *vs*. ASAL(no lice)], possibly due to lice-induced tissue-level changes in the skin (e.g., increased angiogenesis). The ASAL+lice co-stimulation (i.e., COS list) had additive and synergistic effects on the induction of genes involved in innate (e.g., additive: *tlr13*, *clec1a*, *mbl2b*; synergistic: *hampa*, *alox5ap*) and adaptive (e.g., additive: *bcl6*; synergistic: *il4r*) immune responses, and induced several genes related to wound healing (e.g., hemostasis) and antibody formation.

Experiment replication –preferably with infected and non-infected groups housed in the same facility– and histological analyses in the future will help validate the present study’s findings. Furthermore, salmonid species such as Atlantic salmon show a high retention rate of paralogues from a whole-genome duplication event ~80 Mya (171). This fact represents both an opportunity to advance in evolutionary physiology knowledge and a challenge for interpreting transcriptomics results due to functional divergence between duplicated genes in Atlantic salmon. The differential regulation of *mbl2a* (lice-inducible) and *mbl2b* (ASAL-inducible) might be a potential example of functional specialization of two gene copies. The present microarray results may have been influenced by cross-hybridization between paralogous transcripts. Nevertheless, microarray hybridization and paralogue-specific qPCR data showed a high correlation, thus proving the robustness of the study’s claims.

As one of the first transcriptomics studies in the field of co-infection in Atlantic salmon, the present study may serve as a reference for future research with sea lice-infected salmon challenged with other vaccines, PAMPs, or live pathogens. Furthermore, it provides candidate gene biomarkers and putative biological processes responding to sea lice and bacterial single- and co-infection in the skin of Atlantic salmon. Future studies and industrial applications may take advantage of the knowledge generated by this study and evaluate the potential of bacterial compounds and extracts as supplements for clinical feeds and vaccine adjuvants for fish. In sum, these results contribute to improving our understanding of the molecular mechanisms governing the Atlantic salmon’s skin response to sea lice and bacteria co-infection and will help in the improvement of disease management in Atlantic salmon aquaculture.

## Data Availability Statement

The datasets presented in this study can be found in online repositories. The names of the repository/repositories and accession number(s) can be found below: https://www.ncbi.nlm.nih.gov/geo/, GSE186292.

## Ethics Statement

The animal study was reviewed and approved by the Animal Care Committee of Memorial University (Animal Care Protocol 17-77-MR).

## Author Contributions

MLR, RT, MF, and AC-S designed the CDRF and JBARB trials. AC-S supervised the conduction of the trials and coordinated the team that participated in the samplings. AC-S, NU, XX, and ZC were involved in the sea lice copepodid challenge. AC-S, NU, XX, TK, SK, JW, ZC, and MLR were part of the sampling team. AC-S, NU, XX, and MLR designed the microarray and qPCR experiments. NU and XX performed the total RNA and all procedures associated with the generation of the microarray data. AC-S, NU, XX, and TK carried out the qPCR analyses. SK helped with the annotation of the 44K microarray. AC-S analyzed and interpreted the microarray and qPCR results. AC-S led the writing of the manuscript. NU, XX, TK, SK, JW, ZC, MF, SS, RT, and MLR provided comments on the first draft of the manuscript. All authors contributed to the article and approved the submitted version.

## Funding

This study was part of the Integrated Pathogen Management of Co-infection in Atlantic Salmon (IPMC) project (Genomic Applications Partnership Program, GAPP #6607), which was funded by the Government of Canada through Genome Canada and Genome Atlantic. The IPMC project was also funded by InnovateNL (Government of Newfoundland and Labrador Department of Tourism, Culture, Industry and Innovation; Leverage R&D award #5401-1019-108), and EWOS Innovation (now part of Cargill, Incorporated). Furthermore, MLR’s research program is supported by a Natural Sciences and Engineering Research Council of Canada (NSERC) Discovery Grant (341304-2012 and 2020-04519), and Ocean Frontier Institute through an award from the Canada First Research Excellence Fund. The publication fee for this article was funded by MLR's NSERC Discovery Grant (2020-04519). AC-S and NU were the recipients of a Mitacs Accelerate Postdoctoral Fellowship during the conduction of the trials. SK’s salary was funded by the Ocean Frontier Institute.

## Conflict of Interest

RT and SS are former and current employees of Cargill Inc., respectively, but did not participate in the microarray/qPCR study design, the result interpretation, and the decision to submit the manuscript for publication. NU participated in this study as a postdoctoral fellow at Memorial University but was recently employed by Aquabounty Canada Inc.

The remaining authors declare that the research was conducted in the absence of any commercial or financial relationships that could be construed as a potential conflict of interest.

## Publisher’s Note

All claims expressed in this article are solely those of the authors and do not necessarily represent those of their affiliated organizations, or those of the publisher, the editors and the reviewers. Any product that may be evaluated in this article, or claim that may be made by its manufacturer, is not guaranteed or endorsed by the publisher.
