# Supplementary material for: Interacting Effects of Sea Louse (Lepeophtheirus salmonis) Infection and Formalin-Killed Aeromonas salmonicida on Atlantic Salmon Skin Transcriptome
Source: Front Immunol. 2022 Mar 24;13:804987. doi: 10.3389/fimmu.2022.804987 (PMC8987027; doi:10.3389/fimmu.2022.804987)
Supplement: Supplementary file 1 [file DataSheet_1.docx]

**Supplementary Methods**

**Animals**

Atlantic salmon smolts were transported from a local salmon farm to the Dr. Joe Brown Aquatic Research Building [JBARB, Ocean Sciences Centre (OSC), Memorial University, NL, Canada]. Smolts were held in 3,800-L fiberglass tanks until transferred to the 620-L tanks used in the trials. While in the 3,800-L tanks, the salmon were PIT (passive integrated transponder; Easy AV, Avid Identification Systems, Norco, CA, USA)-tagged for growth monitoring and identification during experimentation. When they reached the desired initial weight [238.9 ± 45.2 g, mean weight ± standard deviation (SD)], salmon were randomly distributed into 2 and 4 tanks at JBARB and the Cold-Ocean Deep-Sea Research Facility (CDRF, OSC), respectively. All tanks were connected to the same flow-through seawater system (12 L/min), and monitored daily for water quality. The seawater was pumped from Logy Bay (NL) and subjected to filtration, temperature adjustment, and UV treatment before reaching header tanks inside JBARB; then, it was distributed to the JBARB tanks and the CDRF. Water from JBARB is UV-treated and oxygenated again inside CDRF, before reaching the CDRF's tanks. CDRF has an Aquatic Containment Level 3-certified (Canadian Food Inspection Agency) biocontainment facility equipped with the same 620-L fiberglass tanks used at JBARB. The photoperiod was 24-h light in both buildings.

Fish were fed with EWOS Dynamic S feed (5 mm; 27% fat, 46% protein) using automatic feeders (AVF6 Vibratory Feeder; Pentair Aquatic Eco-Systems, Inc., Nanaimo, BC, Canada) at a daily ration of 1% body weight. Water temperature and dissolved oxygen levels were relatively constant (~10-11 ºC and ≥ 10 mg/L) throughout the acclimation and trial periods. Thirty-five salmon were allocated in each of the tanks at CDRF and 15 in each of the tanks at JBARB. CDRF's fish were destined for the *Lepeophtheirus salmonis* challenge trial, and those at JBARB to serve as no-lice infection controls.

**Sea Lice Challenge**

The salmon at CDRF were challenged with *L. salmonis* at the copepodid stage 79 days after being transferred from JBARB. Sea lice copepodids were provided by Huntsman Marine Science Centre (St. Andrews, NB, Canada). The lice were actively swimming when received at the CDRF, and no significant mortality was observed. The salinity of the seawater containing the lice during their transportation –in sealed plastic bags with ~¾ of their volume filled with pure oxygen– was similar to that of CDRF's circulating seawater (~33 ‰), and the dissolved oxygen levels (DO) were 17-21 mg/L. However, the lice were let to acclimate to CDRF's seawater temperature, which was slightly higher than that of the transportation seawater (10.2 ºC *vs.* 9.0 ºC, respectively).

As previously described (34), in preparation for lice exposure, water flow into the tanks was interrupted, and water volume was reduced by 50%. Oxygen was supplied to the water remaining in the tanks using air diffusers to prevent hypoxia. Then, sea lice copepodids were released into the tanks at a ratio of 50 lice/fish and allowed to infect the salmon for 2 h. During the exposure, water DO, and temperature were measured every 10 min. Any decrease in DO level during the challenge was quickly addressed by adjusting the air supply, and remained above 7.1 mg/L and 72% saturation. No DO supersaturation occurred during the challenge. Water temperature increased by 0.4-0.6 ºC on average. No mortalities were observed. After the 2-h exposure period, the water supply was restored.

**Injection Challenge and Sample Collection**

Four weeks after sea lice exposure, when lice were at the pre-adult stage, lice-infected salmon (CDRF) and non-infected salmon (JBARB) were fasted for 24 h and then subjected to an intraperitoneal (IP) injection of either phosphate-buffered saline (PBS; Gibco/ThermoFisher Scientific, Mississauga, ON, Canada), a solution of polyriboinosinic polyribocytidylic acid (Sigma-Aldrich, Oakville, ON, Canada), or a suspension of formalin-killed *Aeromonas salmonicida* (ASAL) (Figure 1A). For each tank at CDRF, 6 fish were injected with PBS, 6 with pIC, and 6 with ASAL at 1 μL/g of fish (wet mass). For each tank at JBARB, 4 fish were injected with PBS, 4-5 fish with pIC, and 4-5 fish with ASAL. The pIC solution was prepared in ice-cold, 0.2 μm-filtered PBS at 2 μg/μL. As described in Hori et al. (29), the ASAL suspension was prepared by pelleting (centrifugation at 2,000 g for 10 min at 4 ºC) and washing with ice-cold, 0.2 μm-filtered PBS a commercial vaccine for typical *A. salmonicida* (Furogen dip, Novartis Canada, Charlottetown, PE, Canada). The pelleting/washing was performed three times, and after the third wash, the pelleted bacterin was suspended in ice-cold, 0.2 μm-filtered PBS at an optical density of 1.0 at 600 nm wavelength.

The tank order for the IP-injection challenge was randomized. Immediately after being netted and before injection, salmon were lightly anesthetized via immersion in seawater baths with 50 mg/L MS-222 (Syndel Laboratories, Vancouver, BC, Canada). Lice-infected salmon at CDRF were individually anesthetized in 10 L of MS-222 seawater solution to recover any louse detached during the procedure. Non-infected salmon at JBARB were netted and anesthetized in groups of 3 in 30 L of MS-222 seawater solution with air supply. Once anesthetized (within 1-2 min after exposure to the anesthetic), each fish was assigned and IP-injected with one of the preparations (i.e., PBS, pIC, and ASAL). After injection, lice-infected salmon (CDRF) were returned to their original tanks and allowed to recover; injected salmon recovering from the anesthesia were separated from those to be injected using a tank divider –a net mounted on a rigid plastic frame to segregate fish without affecting seawater circulation. Non-infected IP-injected salmon (JBARB) were allowed to recover in 620-L auxiliary tanks connected to the flow-through water system before returning to their original tank.

At 24 h post-injection, salmon were euthanized by immersion in a seawater bath with 400 mg/L MS-222 and dissected for tissue sample collection. The tank sampling order was the same as in the IP-injection challenge. Euthanized salmon were identified using a PIT-tag reader (AVID Power Tracker V, Avid Identification Systems, Calgary, AB, Canada), weighed, and fork length-measured. Two 1-cm^2^ dorsal skin samples were taken from every lice-infected salmon (CDRF; preferably from the left side –samples for other analyses were taken from the right side): one sample around a louse attachment site and another sample from an adjacent intact skin area (i.e., no lice attached or damaged) (Figure 1B). In some cases, dorsal skin samples were taken from the right side of the animal because no adequate louse attachment site was found on the left side. Dorsal skin samples (also 1 cm^2^) from non-infected salmon (JBARB) were always taken from the left side of the animal, starting under the end of the dorsal fin and above the lateral line. All samples were cleaned of any skeletal muscle tissue. All collected samples were immediately flash-frozen with liquid nitrogen and stored at -80 ºC until processed for RNA extraction. Once sample collection for a given lice-infected salmon was complete, the lice remaining on the animal were counted. Total lice count was calculated as the sum of the lice detached during the IP-injection and sampling procedures and those still attached to the salmon.

**Network and Gene Ontology Enrichment Analyses**

Gene ontology (GO) term enrichment analyses (GTEA) were conducted for each DEP list using ClueGO (40) plugin in Cytoscape (v3.5.1) (41). This analysis disregarded DEP redundancy (i.e., multiple probes annotated as the same gene); it only considered the differentially expressed genes (DEGs) putatively represented by the DEP lists. Right-sided hypergeometric tests (i.e., for GO term over-representation) were performed using the human Gene Ontology database (UniProt: 27.02.2019) for Biological Processes (BPs), with an adjusted p-value cut-off level (Benjamini-Hochberg test) of 0.05. The entire 44K salmon array was used as the reference gene list. ClueGO linked the over-represented GO terms using kappa statistics (42), thus generating GO term networks. Cohen's kappa coefficients were calculated for each term-term relationship based on the shared genes between them. The obtained term-term kappa coefficients were also used to define groups of highly connected terms within the GO term networks. The kappa coefficient threshold for the analysis was 0.4. The relative frequency of up-regulated and down-regulated DEGs was used to calculate the z-score (43) of each GO term arising from the enrichment analysis. The formula used to calculate the z-score was:$zscore= \frac{\left( up-down \right)}{\sqrt{total}}$, where *up* and *down* are the number of up- and down-regulated DEGs, respectively, and *total* the total count of DEGs.

The over-represented GO terms were classified, using Gene Ontology Browser (http://www.informatics.jax.org), into 4 functional themes: 1) metabolic processes; 2) cellular processes; 3) immune/stress processes; and 4) development/healing processes. The GO terms were classified based on the biological process to which they were related and/or their parent terms (especially for highly-specific terms). Briefly, GO terms of biological processes and pathways associated with metabolism-related processes and pathways and/or that have the parent term "GO:0008152 metabolic process" were classified as "metabolic process". GO terms associated with responses to stress (e.g., response to heat) and immune (e.g., response to bacterium) stimuli fell within the "immune/stress process" theme. GO terms related to tissue development and healing and/or derived from the parent term "GO:0032502 developmental process" were classified as "development/healing process". The theme "cellular process" grouped all those GO terms not classifiable in any of the previous and derived from "GO:0009987 cellular process". Some GO groups comprised terms from different themes; in such cases, the group is colored according to the theme with the highest number of GO terms.

**qPCR Analyses**

First-strand cDNA templates were synthesized in 20 μL reactions from 1 μg of DNaseI-treated, column-purified total RNA, dNTPs (0.5 mM final concentration for each dNTP; Invitrogen, Thermo Fisher Scientific), random primers (250 ng; Invitrogen), DTT (10 mM final concentration; Invitrogen), first-strand buffer (1X final concentration; Invitrogen) and M-MLV reverse transcriptase (200 U; Invitrogen) at 37 ºC for 50 min.

The qPCR amplifications were conducted in 384-well format on a ViiA 7 Real-Time PCR system (Applied Biosystems/Life Technologies, Foster City, CA, USA), and followed the Minimum Information for Publication of qPCR Experiments (MIQE) guidelines (44). Reaction mixtures (13 μL) consisted of 1X Power SYBR Green PCR Master Mix (Applied Biosystems/Life Technologies), 50 nM of both the forward and reverse primers, and diluted cDNA template (5 ng input total RNA). The qPCR program was 1 cycle of 50 °C for 2 min, 1 cycle of 95 °C for 10 min, and 40 cycles of 95 °C for 15 s and 60 °C for 1 min, with fluorescence detection at the end of each 60 °C step. qPCR reactions for primer quality testing were performed in technical duplicates. The qPCR analyses of the transcript levels of the selected genes of interest (GOIs) and candidate normalizer genes were carried out in technical triplicates. No-template controls were included for every qPCR analysis.

Forty-two microarray-identified GOIs were qPCR analyzed to confirm the microarray results (see Statistical Analyses for more information). GOIs were selected based on their induction/repression intensity, their involvement in relevant over-represented biological processes, and/or because their expression levels were significantly correlated with total lice load (i.e., sum of attached and detached lice counts). For those GOIs for which there were no qPCR primers previously developed, cDNA sequences from NCBI non-redundant nucleotide (nt) and expressed sequence tags (EST) databases were compiled using BLASTn searches and 44K probe information (i.e., contigs and 60mer sequences). BLASTn-searches were performed between August and November 2020. Possible microarray-identified paralogue cDNA sequences were aligned using Vector NTI (Vector NTI Advance 11, Life Technologies) to determine identity between paralogues and to find suitable regions (i.e., with at least 3 bp difference) for paralogue-specific qPCR primer design. New primers were designed using Primer 3 v.0.4.0 software [available at (<http://bioinfo.ut.ee/primer3-0.4.0/)>]. Primer pairs were quality-tested for single-product amplification (via dissociation curve analysis), absence of primer-dimers in the no-template control, and correct PCR product size [through comparison with 1 kb Plus DNA Ladder (Invitrogen/Life Technologies) using 2% agarose gel electrophoresis]. Furthermore, amplification efficiency (45) was analyzed using 5-point 1:3 dilution series using cDNA synthetized from ASAL/lice and PBS/no lice RNA sample pools. The dilution series started with cDNA representing 10 ng of input total RNA. All information concerning primer sequences and quality-check results is shown in the Supplementary Table S1.

Five candidate normalizer genes were tested for mRNA level stability across injection/infection groups. These genes were *60S ribosomal protein L32* (*rpl32*), *elongation factor 1-alpha 1* (*ef1a1*), *polyadenylate-binding protein, cytoplasmic 1* (*pabpc1*), *eukaryotic translation initiation factor 3 subunit D* (*eif3d*), *ATP binding cassette sub-family f member 2* (*abcf2*). These candidate normalizer genes were selected based on previous experience with infected or PAMP-challenged Atlantic salmon (34, 46). *rpl32* and *pabpc1* were chosen as the most stably expressed based on geNorm analyses [M-values 0.160 and 0.158, respectively; qBASE plus, Biogazelle NV, Belgium (48)].

The relative quantity (RQ) of each qPCR-analyzed GOI was calculated using a qBase relative quantification framework (49, 50) through normalization to *rpl32* and *pabpc1*, with amplification efficiencies incorporated. The RQ values of each GOI was calibrated to the sample that had the lowest normalized gene expression (i.e., assigned an RQ value = 1.0).
