# Supplementary figures and images for: Interacting Effects of Sea Louse (Lepeophtheirus salmonis) Infection and Formalin-Killed Aeromonas salmonicida on Atlantic Salmon Skin Transcriptome
Source: Front Immunol. 2022 Mar 24;13:804987. doi: 10.3389/fimmu.2022.804987 (PMC8987027; doi:10.3389/fimmu.2022.804987)

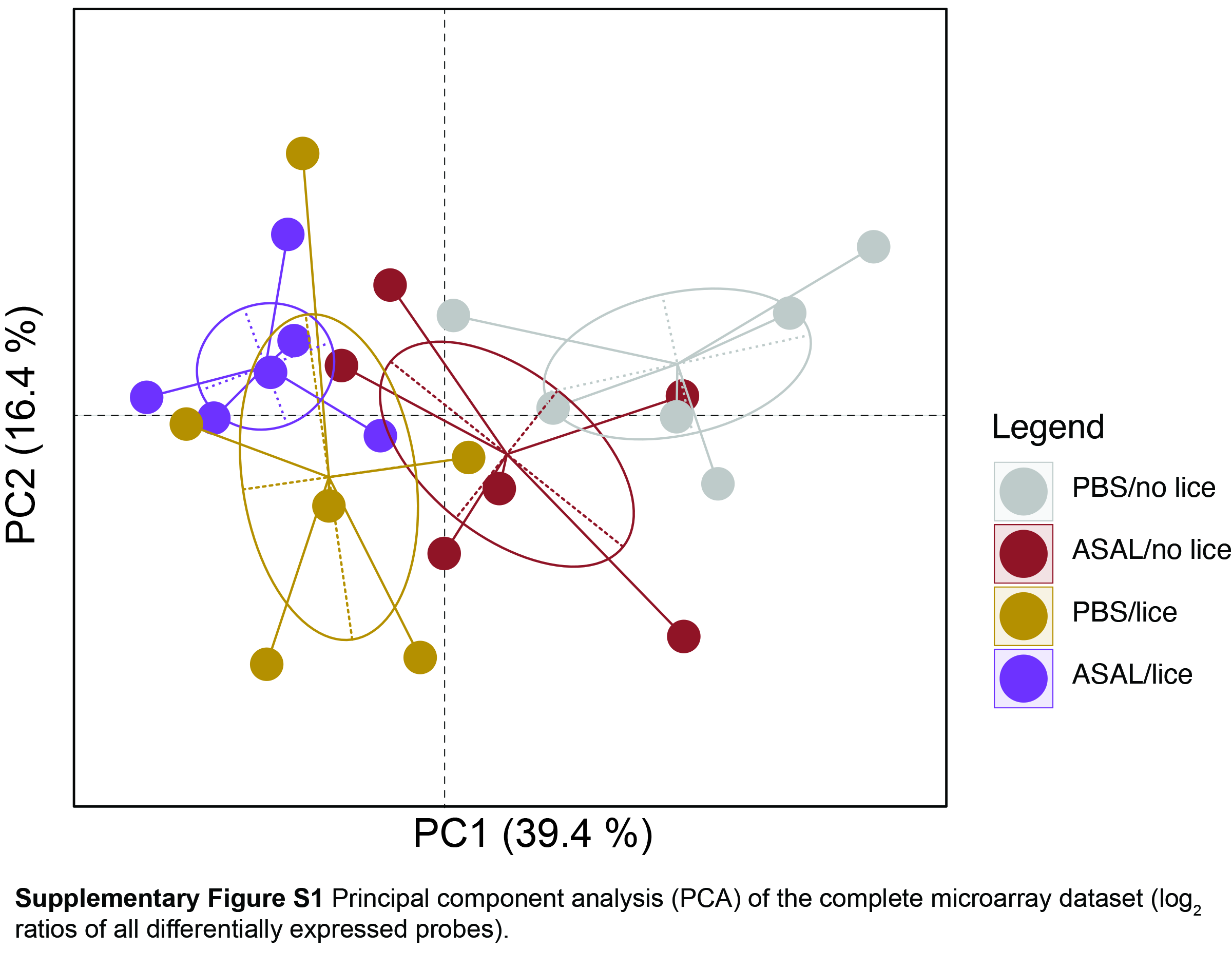

Supplement: Supplementary file 2 [file Image_1.tif]

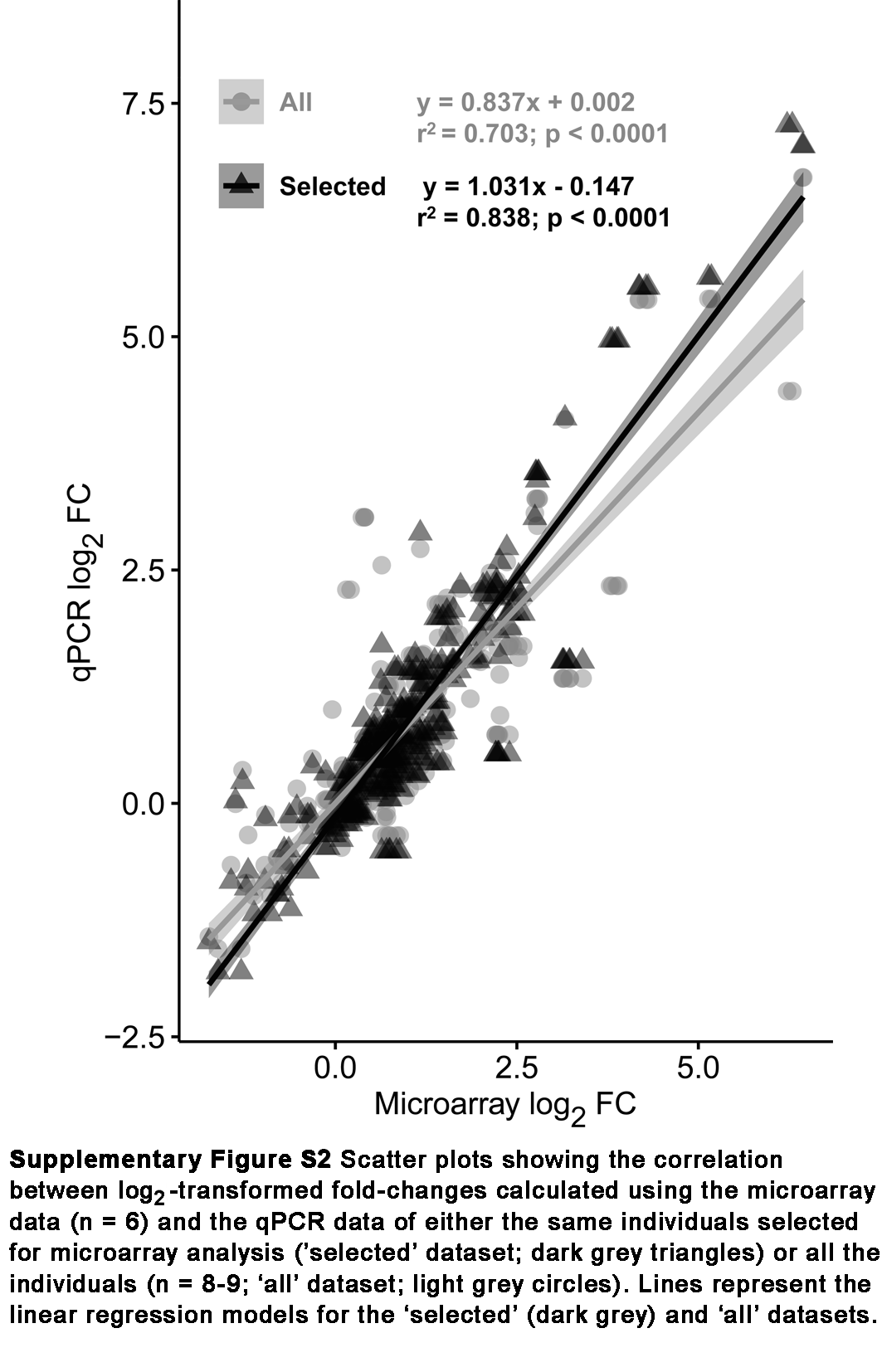

Supplement: Supplementary file 3 [file Image_2.tif]
